# Training in the use of intrapartum electronic fetal monitoring with cardiotocography: systematic review and meta‐analysis

**DOI:** 10.1111/1471-0528.16619

**Published:** 2021-01-22

**Authors:** S Kelly, P Redmond, S King, C Oliver‐Williams, G Lamé, E Liberati, I Kuhn, C Winter, T Draycott, M Dixon‐Woods, J Burt

**Affiliations:** ^1^ THIS Institute (The Healthcare Improvement Studies Institute), Department of Public Health and Primary Care University of Cambridge Cambridge UK; ^2^ School of Population Health and Environmental Sciences King’s College London London UK; ^3^ Independent consultant Cambridge UK; ^4^ Cardiovascular Epidemiology Unit Department of Public Health and Primary Care University of Cambridge Cambridge UK; ^5^ Homerton College University of Cambridge Cambridge UK; ^6^ PROMPT Maternity Foundation Southmead Hospital Bristol UK; ^7^ Translational Health Sciences University of Bristol Bristol UK; ^8^ Present address: Laboratoire Genie Industriel CentraleSupélec Université Paris‐Saclay Gif‐sur‐Yvtte France

**Keywords:** Caesarean, clinical outcome, fetal heartbeat monitoring, fetal heartrate monitoring, health personnel, intervention, intrapartum, Kirkpatrick model, mixed methods, neonatal, observational study, pregnancy

## Abstract

**Background:**

Sub‐optimal classification, interpretation and response to intrapartum electronic fetal monitoring using cardiotocography are known problems. Training is often recommended as a solution, but there is lack of clarity about the effects of training and which type of training works best.

**Objectives:**

Systematic review of the effects of training healthcare professionals in intrapartum cardiotocography (PROSPERO protocol: CRD42017064525).

**Search strategy:**

CENTRAL, Cochrane Library, MEDLINE, EMBASE, PsycINFO, British Nursing Database, CINAHL, ERIC, Scopus, Web of Science, ProQuest, grey literature and ongoing clinical trials were searched.

**Selection criteria:**

Primary studies that reported impact of training healthcare professionals in intrapartum cardiotocography. Title/abstract, full‐text screening and quality assessment were conducted in duplicate.

**Data collection and analysis:**

Data were synthesised both narratively and using meta‐analysis. Risk of bias and overall quality were assessed with the Mixed Methods Appraisal Tool and GRADE.

**Main results:**

Sixty‐four studies were included. Overall, training and reporting were heterogeneous, the outcomes evaluated varied widely and study quality was low. Five randomised controlled trials reported that training improved knowledge of maternity professionals compared with no training, but evidence was of low quality. Evidence for the impact of cardiotocography training on neonatal and maternal outcomes was limited, showed inconsistent effects, and was of low overall quality. Evidence for the optimal content and method of delivery of training was very limited.

**Conclusions:**

Given the scale of harm and litigation claims associated with electronic fetal monitoring, the evidence‐base for training requires improvement. It should address intervention design, evaluation of clinical outcomes and system‐wide contexts of sub‐optimal practice.

**Tweetable abstract:**

Training in fetal monitoring: systematic review finds little evidence of impact on neonatal outcomes.

## Tweetable abstract

Training in fetal monitoring: systematic review finds little evidence of impact on neonatal outcomes.

## Introduction

Preventable harm related to childbirth can be catastrophic for women, children and families.^1^ Intrapartum fetal monitoring is intended to identify fetal compromise and facilitate appropriate action in response.^2^ However, suboptimal practice, particularly in relation to electronic fetal heart rate monitoring (EFM) using cardiotocography (CTG) in labour, is a known problem and remains frequently cited in successful obstetric malpractice claims.^3^, ^4^ Training of maternity personnel remains the most frequently recommended intervention to improve EFM.^5^, ^6^ Despite its ubiquity, it is unclear whether CTG training improves birth outcomes, and, if so, which form of training is most effective.^7^, ^8^

A previous systematic review of studies published between 1978 and 2009, based on searches of Medline alone, located only 20 studies.^9^ Since 2009, many more studies on CTG training have been published, and formal methods for review have become better established.^10^, ^11^ We aimed to use up‐to‐date and methodologically robust systematic review methods to examine the effects of training in intrapartum CTG and to assess evidence for optimal methods of training.

## Methods

We conducted a systematic review and, in selected papers, a meta‐analysis. We used PRISMA and MOOSE guidelines to guide reporting.^12^, ^13^

### Eligibility criteria

All primary research studies that examined the impact of intrapartum CTG training for healthcare professionals were eligible for inclusion, irrespective of study design (quantitative or qualitative), language, date, length of follow up, or publication status (conference abstracts were included). Full eligibility criteria were:

- Population: All studies involving healthcare professionals receiving intrapartum CTG training.
- Intervention/exposure: All studies examining the impact of CTG training for healthcare professionals, including those where CTG training was included as one component of a more complex intervention, with no restrictions on the length of training or the method(s) by which training was delivered. Studies referring to EFM without explicitly referencing CTG were included, as the terms are often used interchangeably.^14^, ^15^
- Comparators: Eligible comparator groups (where applicable) included no intervention, usual practice, a different type of training, or different components of training.
- Outcomes: As we anticipated that the studies would examine a wide range of outcomes, we did not pre‐specify detailed outcomes. We instead described the full range reported in the included studies.

## Information sources and search strategy

The search strategy, developed with an experienced information specialist (IK), included MeSH headings, text words and synonyms for CTG and training (Appendix S1). We searched the following databases from inception to the end of October 2019: Cochrane Central Register of Controlled Trials (CENTRAL), Cochrane Library, MEDLINE, EMBASE, PsycINFO, British Nursing Database, CINAHL, ERIC, Proquest Dissertations and Theses, Scopus, Web of Science. The MEDLINE search strategy was translated for other databases using appropriate syntax and vocabulary.

Grey literature searches were conducted in Open Grey, Grey Literature report and NICE Evidence Search. Searches for registered ongoing trials were conducted in the WHO International Clinical Trials Registry Platform search portal and ClinicalTrials.gov. We additionally reviewed reference lists of all included studies and relevant systematic reviews, and contacted relevant experts.

## Study selection

Title/abstract and full‐text screening were conducted independently by two reviewers. Differences were resolved by discussion or with a third reviewer.

## Data extraction

Two reviewers independently extracted data, using a predefined data collection template, with disagreements resolved by discussion. Data extracted included participants, country, study design, description of the training intervention and comparator, setting and context, inclusion and exclusion criteria outcomes, outcome measurement, results and funding. Where necessary, study authors were contacted to clarify information or seek unpublished results/data.

We tabulated and examined all pre‐intervention and post‐intervention results (sample sizes, means, proportions, 95% CI) for each group within each outcome, and differences between groups, where available.

## Assessment of risk of bias

### Individual studies

Risk of bias was assessed independently by two reviewers using the Mixed Methods Appraisal Tool (MMAT),^16^ a validated tool developed for the appraisal of diverse study types. MMAT classifies study designs as: (1) qualitative, (2) quantitative randomised, (3) quantitative non‐randomised (non‐randomised intervention studies and observational cohort studies), (4) quantitative descriptive (e.g. surveys, case series, incidence or prevalence study) or (5) mixed methods.

For each study design, the MMAT assesses several domains of risk of bias criteria, each assessed as low, high or unclear risk of bias, but does not provide an overall summary risk of bias estimate across domains. To facilitate the next stage of GRADE (the Grading of Recommendations, Assessment, Development and Evaluation^17^) assessment, we devised a summary estimate for each study based on a global assessment across the MMAT domains. Each study overall was classified as low, high or unclear risk of bias.

### Across studies

We used GRADE to assess the quality of the overall body of evidence for each outcome using GRADEpro software.^18^ GRADE categorises the overall evidence as high, moderate, low or very low. The method initially classifies randomised controlled trials (RCTs) as ‘high quality’, but in some instances GRADE requires downgrading of evidence if there is serious risk of bias, imprecision, inconsistency of results, indirectness or publication bias. Non‐randomised interventions and observational studies are initially assessed as ‘low quality’, which can be upgraded to a higher level for large effect size or a dose–response relationship.

## Data synthesis

Data were organised into three broad groups reflective of the underlying studies:

1. Comparisons of different methods of intrapartum CTG training
2. Evaluations of the impact of intrapartum CTG training alone
3. Evaluations of intrapartum CTG training as one part of a larger intervention.

The previous review in this area^9^ used the Kirkpatrick four‐level model of training^19^ to guide data organisation and synthesis (Table 1). We used the same approach, classifying outcomes as relating to: learners’ reactions, learning as a result of training, behaviours following training, and clinical outcomes. Ambiguities about classification were resolved through review team discussion.

**Table 1:** Kirkpatrick levels of training evaluation

Level	Description
Reaction (Level 1)	Participants' reactions to and opinions about CTG training they received
Learning (Level 2)	The extent to which participants acquired new knowledge or skills as a result of CTG training
Behaviour (Level 3)	The extent to which learning as a result of CTG training was applied in the workplace
Results (Level 4)	The assessment of outcomes that could be attributed to CTG training, such as changes in patient outcomes or resource use

We pooled data in a meta‐analysis (using Cochrane revman 5∙3^20^) where studies reported similar study design, outcomes, outcome measures and measure of association. Because of heterogeneity of study populations, interventions and designs, we used a random effects model. Outcomes were measured in different ways, so standardised mean difference expressing the size of the intervention effect relative to the variability for each study,^11^ or risk ratios, were used as the summary statistics. Pooled estimates with 95% CI were calculated using generic inverse variance. Statistical heterogeneity was measured using the *I* ^2^ statistic.^11^, ^21^, ^22^

### Protocol and registration

The protocol was registered on PROSPERO (CRD42018082567). Minor differences from protocol are described in Appendix S2. There was no patient or public involvement in this study. No core outcome set was used.

## Results

### Study selection and characteristics

**Figure 1:**
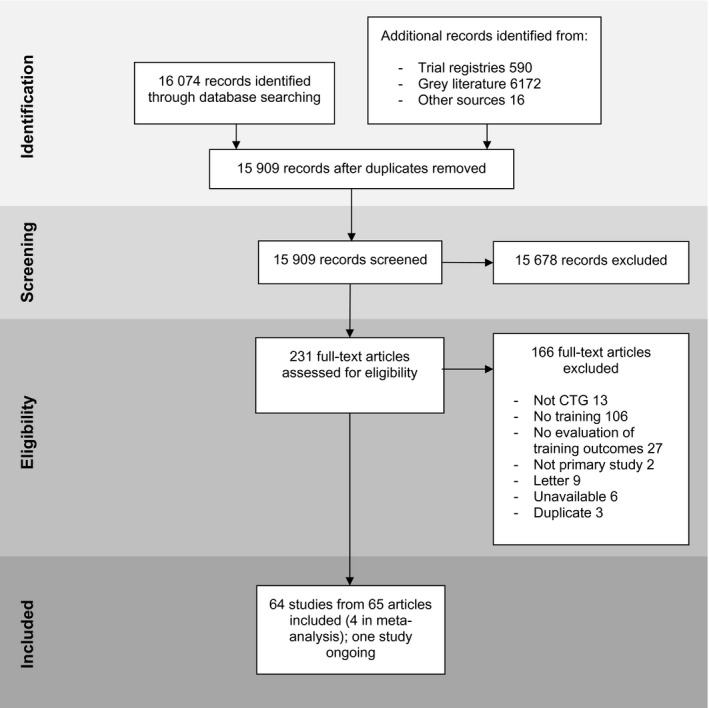
PRISMA flow diagram.

We identified 64 studies that met our inclusion criteria (Figure 1) reported across 65 papers (two papers reported different outcomes from the same study).^23^, ^24^, ^25^, ^26^, ^27^, ^28^, ^29^, ^30^, ^31^, ^32^, ^33^, ^34^, ^35^, ^36^, ^37^, ^38^, ^39^, ^40^, ^41^, ^42^, ^43^, ^44^, ^45^, ^46^, ^47^, ^48^, ^49^, ^50^, ^51^, ^52^, ^53^, ^54^, ^55^, ^56^, ^57^, ^58^, ^59^, ^60^, ^61^, ^62^, ^63^, ^64^, ^65^, ^66^, ^67^, ^68^, ^69^, ^70^, ^71^, ^72^, ^73^, ^74^, ^75^, ^76^, ^77^, ^78^, ^79^, ^80^, ^81^, ^82^, ^83^, ^84^, ^85^, ^86^ One relevant study protocol was not included because the study is still ongoing.^87^ Study designs, as classified by MMAT criteria, included: 13 RCTs, 40 quantitative non‐randomised studies (from 41 papers) and 11 quantitative descriptive studies. Two studies reported some textual information using open‐ended questions on questionnaires.^52^, ^80^ No relevant qualitative studies were found. Full data tables for all included studies can be found in Appendix S3.

### Settings

Studies were conducted in the USA (*n* = 27), the UK (*n* = 14), Sweden (*n* = 3), Australia (*n* = 3), France (*n* = 2) and Taiwan (*n* = 2), with one study each in Portugal, Ireland, Saudi Arabia, Canada, Italy, the Netherlands, Japan, China, Denmark, India, Spain and Turkey, and one jointly between Australia and New Zealand.

Twenty‐one studies involved delivery of training in labour or maternity units; 15 in universities, classrooms or simulation centres; eight in university hospitals; and six in general hospitals. In 11 studies, it was unclear in what setting the training took place. Three studies reported analyses of national data sets.

### Participants

Twenty‐nine studies reported diverse training participants often collectively referred to as ‘maternity care providers’, covering various combinations of healthcare professionals and students. Eleven further studies focused on nurses alone; three on residents/attendings in obstetrics; three on medical students; seven on nursing/midwifery students and three on a mix of medical and nursing students. Three studies reported that all organisational staff or all maternity care staff, including clinical leaders, were involved in training. In five studies, the staff targets of the intervention were unclear.^45^, ^62^, ^63^, ^71^, ^73^

The actual numbers participating in each of the studies were often not reported or were unclear.^26^, ^41^, ^69^, ^70^, ^71^, ^72^, ^73^, ^74^, ^77^, ^78^, ^84^ Where participant numbers were reported, they varied from six^51^, ^75^ to 4439^81^ individuals.

### Overall certainty of evidence across studies: GRADE assessments

A summary of overall GRADE assessments is provided in Tables S1 and S2. Full details of GRADE assessments, including reasons for upgrading or downgrading the level of evidence, are shown in Appendix S4. The certainty of the available evidence overall based on GRADE is low or very low across all categories of training and outcomes.

### Risk of bias

**Figure 2:**
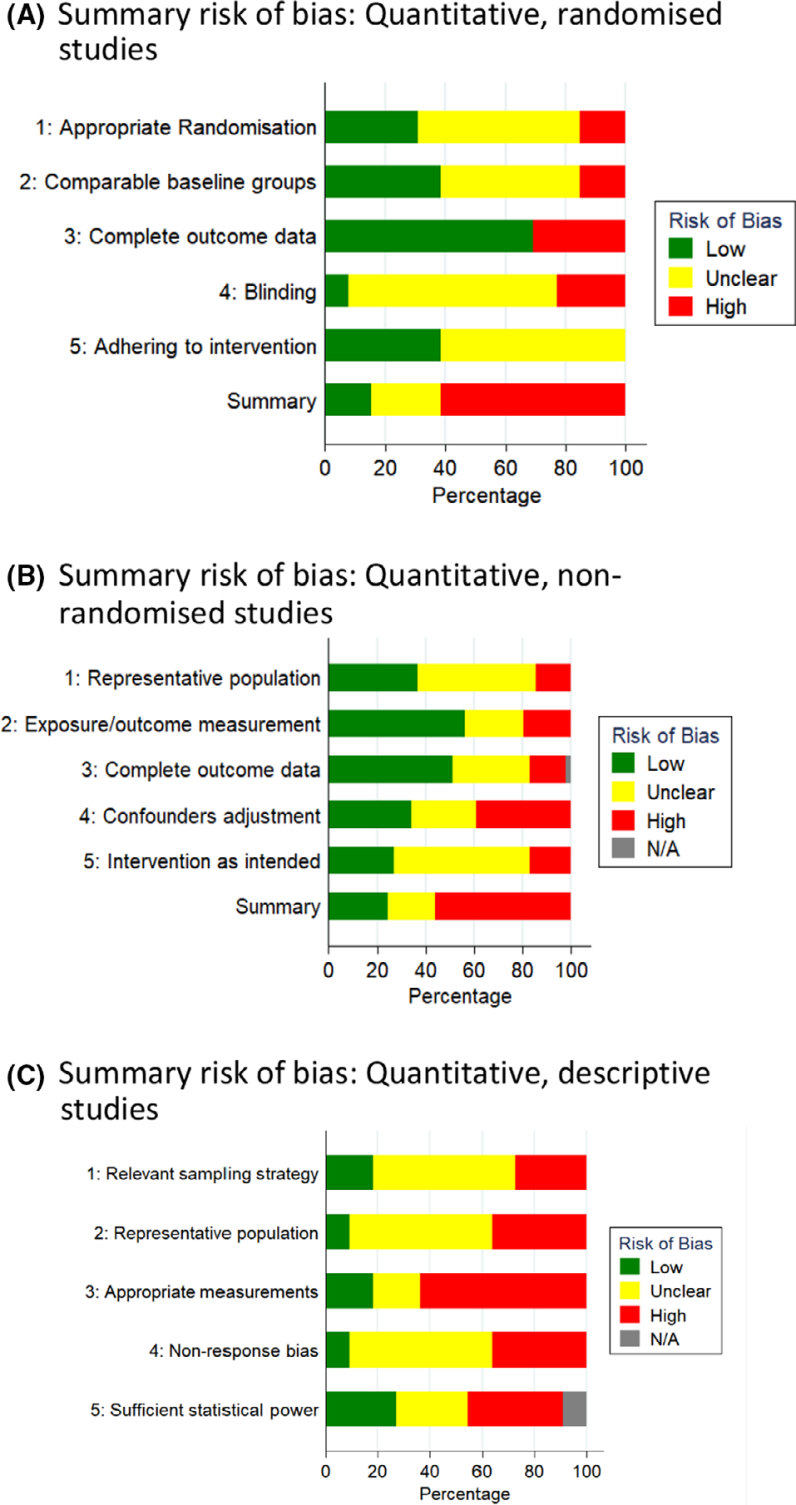
Summary risk of bias plots (MMAT criteria). (A) Summary risk of bias: quantitative, randomised studies. (B) Summary risk of bias: quantitative, non‐randomised studies. (C) Summary risk of bias: quantitative, descriptive studies.

Summary risk of bias assessments for included studies, using the MMAT criteria, are shown in Figure 2 (for risk of bias for individual studies, see Appendix S5). Of the 13 RCTs included in the review, eight were at high risk of bias,^24^, ^25^, ^29^, ^30^, ^32^, ^33^, ^34^, ^35^ for three it was unclear^23^, ^26^, ^27^ and two had low risk of bias.^28^, ^31^ Inappropriate or insufficient details of randomisation and blinding were of particular concern (Figure 2; Appendix S5).

Of the 40 quantitative non‐randomised studies, 23 were at high risk, 8 at unclear risk, and 10 at low risk of bias. Particular areas of concern were the methods of measurement used, poor reporting of outcomes and lack of adjustment for potential confounders. Of the 11 quantitative descriptive studies, four had high risk of bias, three were unclear and four had low risk of bias.

### Publication bias

Though 13 RCTs overall were included, they reported diverse training approaches, outcomes and outcome measurement. Given the heterogeneity, we therefore did not formally assess publication bias using funnel plots, as they are not usually recommended for fewer than ten RCTs.^11^ Some studies (*n* = 11, Appendix S3) were published as conference abstracts with no subsequent publication of a full peer‐reviewed paper. This may imply some publication bias.

### Synthesis of results

#### Content and methods for delivering intrapartum CTG training

Descriptions of the content of and delivery of CTG training were generally poor, failing to conform to good reporting practice.^88^ It was typically difficult to distinguish different components (such as training on CTG trace interpretation versus response to concerning traces) or the relative weight given to the components. Where content and methods for training delivery were described, the approaches reported were diverse, often multi‐faceted, including traditional classroom‐based teaching and lectures, online e‐learning, simulation training, and algorithms or multiple components (Appendix S6).

Only nine studies specifically compared different methods of training (Table S1). All reported knowledge outcomes or interpretation skills as assessed through test scores (Kirkpatrick level 2).

Of these, five studies compared one method of training delivery with another (Table S1),^34^, ^35^, ^54^, ^85^, ^89^ the overall certainty of evidence was low. One study found that simulation training led to improved skills in EFM interpretation compared with traditional lecture‐based training,^85^ although risk of bias was unclear. Four compared traditional methods of teaching (lectures or seminars) with technology‐assisted instruction (e.g. via computers, videos and mobile phones). Only one of these, a non‐randomised study, found an effect of training: students given an active learning simulation of EFM on a mobile device had significantly better test scores than those who received a reading assignment, but risk of bias was high.^54^

Four studies, all RCTs, examined different components of training programmes (Table S1).^30^, ^31^, ^32^, ^33^ Only one of these – a comparison of computer‐assisted learning alone with this intervention plus a tutorial – found evidence for an improvement in EFM knowledge,^30^ but was assessed as having a high risk of bias.

It was not possible to determine the impact of different methods of training on behaviours or neonatal/maternal outcomes as no study reported them.

#### Impact of intrapartum CTG training on reactions, knowledge, behaviours and outcomes

Studies that sought to examine the impact of CTG training, compared with no training, on participants’ reactions, knowledge, behaviours and clinical outcomes are described below in relation to the four Kirkpatrick levels (Table 1).

##### (1) Learners’ reactions to CTG training (Kirkpatrick level 1)

Participants’ reactions to CTG training, including training in workshops and simulations and technologically‐assisted training, were evaluated in eleven studies (two RCTs, two non‐randomised studies and seven quantitative descriptive studies),^23^, ^83^, ^84^ mostly using questionnaires. Although studies reported that participants and facilitators were positive about training, these findings must be interpreted with caution. Many studies gave ill‐defined assessments using phrases such as ‘virtually all’ or ‘almost all’ participants, with limited numerical data. We deemed the certainty of this evidence very low.

##### (2) Learning as a result of CTG training (Kirkpatrick level 2)

Evaluations of the impact of CTG training on participants’ knowledge and skills involved learners’ scores on tests, assessments of inter‐observer agreement on CTG classification, and assessments of post‐intervention performance in simulated scenarios (Table S2).

**Figure 3:**
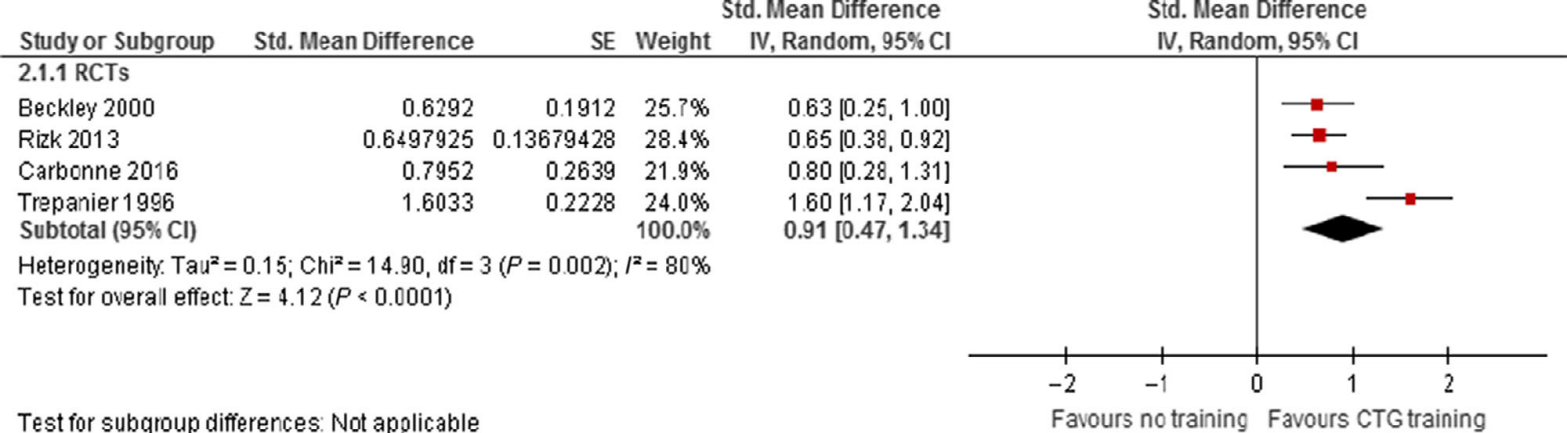
Forest plots: meta‐analysis of RCTs that compared training versus no training on test results. Only an overall sample size was reported in Beckley et al. (2000),^23^ so we divided the sample size in half; given the number of outcomes reported in Rizk and Hafez (2013),^26^ we selected one outcome ‘general knowledge about EFHM’ at 1 month after the intervention to include in the above analysis; for Trépanier et al. (1996),^27^ we selected results from the ‘knowledge test’ immediately after the intervention to include in the above analysis (see Appendix S3 for selection reasons). One further RCT (Devine & Lalor 2006),^25^ could not be included in the above analysis as the authors only reported median and interquartile data (as the data were not normally distributed), meaning that the means and standard deviations needed for meta‐analysis could not be estimated from these data. The results from this trial, however, are consistent with the above (i.e. they demonstrated a statistically significant impact of CTG education on improving test scores measuring knowledge).

Five RCTs used test results to compare the impact on knowledge of CTG training versus no training (Table S2). Training approaches included computer‐assisted teaching (two trials^23^, ^24^); lectures or workshops (two trials^25^, ^27^); or both.^26^ All five RCTs reported a significant positive effect on knowledge following training. Data from four of these five studies could be pooled and demonstrated an effect in favour of CTG training (standardised mean difference 0.91, 95% CI 0.47–1.34; *I* ^2^ 80%, *P* < 0.0001, 487 participants) (Figure 3). Mean and standard deviation (SD) could not be extracted from the fifth study.^25^

One study found a larger effect of training than the other three studies,^27^ and appeared to contribute to the high heterogeneity across the four studies (*I* ^2^ = 80%). In a post‐hoc sensitivity analysis excluding this study, there was no heterogeneity (*I* ^2^ = 0) and a positive effect of training remained (standardised mean difference 0.67 (95% CI 0.46–0.87, *I* ^2^ 0%, *P* < 0.00001, 383 participants). A possible explanation for the heterogeneity is that this study involved a face to face element of training and the others were computer based. Using GRADE, we deemed the certainty of these effect estimates to be low (Appendix S4).

Thirteen further non‐randomised studies evaluated mean test scores, or the proportion of correct answers or improvement in scores (Table S2). Two additional studies solely reported pass rate scores after training. Across these 15 studies, correct test scores ranged from 47 to 64% before training and from 66 to 99% after training; overall improvements ranged from 2 to 44%. This additional evidence generally supports the RCT evidence in suggesting a positive impact of CTG training on knowledge, but the certainty using GRADE was also low.

Three RCTs evaluated healthcare professionals’ ‘performance’ after CTG training (Table S2).^26^, ^28^, ^29^ Though performance was evaluated in different ways in each study, they all focused on new knowledge or skills, so were classified as Kirkpatrick level 2. All three studies involved simulation‐based training. CTG training was, however, the main component of only one study;^26^ it reported an improvement in nurses’ performance score. In the other two studies, CTG training was only one component of wider obstetric training, so the specific effect of CTG training could not be separated.^28^, ^29^ Overall, evidence for the effect of CTG training on participants’ performance in simulated scenarios is of low certainty using GRADE.

Four of the included studies (one non‐randomised controlled trial, two pre‐post studies and a cross‐sectional survey) considered impact of training on inter‐observer agreement on CTG interpretation (Table S2), addressing the known problem of variability in classification of traces.^90^, ^91^, ^92^, ^93^ Where reported, delivery of training involved short‐term classroom and practical sessions,^75^ audit and review,^44^ or online training.^50^ All four reported that training improved inter‐observer agreement. However, only one study reported statistical evidence for improvement.^50^ Using the GRADE approach, we considered the certainty of this evidence to be very low.

##### (3) Behaviours following CTG training (Kirkpatrick level 3)

Changes in behaviour and application of learning following CTG training were assessed in nine non‐randomised studies (four reported as abstracts only) and one descriptive study (Table S2). Diverse outcomes were examined across these studies (Table S2). The studies generally suffered from lack of clarity (e.g. insufficient details of changes from baseline), poor presentation of results (e.g. graphs with no accompanying numerical data), and the use of unvalidated outcome measures.

Overall, there was mixed and inconclusive evidence from these studies. Using GRADE, the overall certainty of these studies was very low.

##### (4) Neonatal/maternal and system outcomes following intrapartum CTG training (Kirkpatrick level 4)

Overall, eight studies examined the impacts of CTG training on key neonatal and maternal outcomes, including: rates of emergency caesarean section, proportion of babies with low Apgar (<5; ≤6; <7) at 5 minutes, neonatal deaths, and hypoxic ischaemic encephalopathy (HIE) (Table S2). Most analysed data from large‐scale cohort studies.

The impact of CTG training on rates of emergency caesarean section was considered in four non‐randomised studies.^38^, ^46^, ^67^, ^68^ Available evidence was inconsistent (Table S2): two studies reported lower rates of emergency caesarean section after CTG training,^38^, ^40^ but the third study reported higher rates.^46^ The fourth study evaluated the implementation of a standardised national CTG education programme comprising an e‐learning programme and 1‐day course. It reported a higher rate of emergency caesarean sections during implementation of training but no evidence of an effect 3 months later.^67^ The overall certainty of evidence was very low, assessed using GRADE.

Evidence for the impact of intrapartum CTG training on the proportion of babies with low Apgar scores (<5; ≤6; <7) at 5 minutes was inconsistent across studies (Table S2). Two studies reported a consistent effect in favour of training^38^, ^46^ and two reported no significant effect of training.^67^, ^68^ Again, using GRADE, the evidence was of low certainty.

Only two studies considered the impact of CTG training on overall neonatal death rates. Both were non‐randomised, assessed as low certainty evidence. Neither study reported a difference in overall neonatal death rates before and after training.^38^, ^40^ One of these reported that hypoxic intrapartum perinatal deaths and neonatal mortality among babies admitted to the neonatal unit were lower after training.^38^

Rates of HIE were reported in six non‐randomised studies that investigated a variety of CTG training approaches, ranging from 1 day to longer programmes (Table S2). Two studies found lower risk of HIE after training,^39^, ^46^ as did one further study that reported hypoxic ischaemia‐related neonatal deaths.^38^ One study found no difference in HIE rates after an intensive training course,^40^ and another additionally reported no effect on rates of moderate/severe HIE.^46^ Full data were not available for two studies, as only abstracts were available.^71^, ^74^ Overall, this evidence was of very low certainty in GRADE assessments.

Not all studies were focused solely on evaluation of CTG training: Nine studies considered CTG training as a component of much wider organisational changes and restructuring (Appendix S3).^37^, ^49^, ^60^, ^62^, ^63^, ^64^, ^70^, ^72^, ^86^ It was not possible to specifically assess the impact of CTG training alone in these studies, and the components and outcomes reported in these organisational interventions varied markedly (Appendix S3). We did not identify any studies that evaluated the impact of CTG training on the use of resources.

## Discussion

### Main findings

This systematic review found that the available evidence on training in the use of intrapartum EFM with CTG is generally of poor quality. Weak study designs characterised most of the 64 studies in this review: only 13 were RCTs; the rest were non‐randomised interventional or observational studies. There is some mostly low‐quality evidence that intrapartum CTG training may improve participants’ knowledge. Evidence of the overall impact of CTG training on clinical outcomes is limited, inconsistent and of low quality, and robust evidence on the optimal content and delivery for CTG training is lacking.

### Strengths and limitations

A strength of this review is its use of methodologically robust and up‐to‐date systematic review methods, including comprehensive searches of a wide range of databases and grey literature, with no restrictions on study type, language or publication date. Using the Kirkpatrick model of training evaluation provided a relevant and flexible framework for the synthesis of complex evidence across participants’ reactions, learning and behaviours, and clinical outcomes.^19^ Limitations of the review include the inability to fully assess publication bias.

### Interpretation

Preventable harm related to childbirth can be catastrophic for women, children and families. Suboptimal interpretation of EFM during labour and subsequent failures to act have been repeatedly identified as the most common contributory factors to poor outcomes in medical negligence claims.^1^ Training in CTG interpretation is one of the most frequently proposed strategies for improving care, even though, as this review shows, the available studies on CTG training do not offer convincing evidence of impact beyond some improvement in participants’ knowledge. Given that, globally, CTG training consumes many thousands of hours of clinical time and remains the major strategy for improving quality and safety of care in relation to EFM, the poor evidence on how, or even whether, these practices can be improved is dismaying.

A first step in addressing these challenges should be commitment to improving the evidence. The available studies vary widely in the training and detail reported, the study designs and the outcomes evaluated. No RCT reported on the impact of training on maternal or fetal outcomes. No study reported a theory of behaviour change or evaluation model. The available studies tend to treat training as a one‐off event, sometimes ‘topped‐up’ at intervals. Optimal frequency of training, and how competence and proficiency can best be assessed over time, remains obscure. The poor quality of the available studies is indicative of the abject status granted to the study of training and education in healthcare^94^ more broadly. Well‐designed trials and evaluative studies should be a priority for future work. They should be theory‐guided,^95^ focused on clinical outcomes, seek to improve understanding of mechanisms of change, and employ established guidelines for reporting.^88^ The design challenges of such trials^9^ might potentially be addressed by simulation‐based study designs.^96^

Improving the quality of new studies will also require attending to basic issues such as what might be the appropriate targets of training. For example, much currently reported training tends to be limited to features of traces and how they should be classified according to predefined criteria, rather than including the context in which the CTG is being used, including the evolution of CTG features, progress in labour and a woman’s individual clinical history. In particular, small‐for‐gestational‐age infants and intrapartum features including maternal pyrexia, uterine activity and meconium independently predict poor neonatal outcomes.^97^ Training in the use of support tools based on national guidance, an emphasis on human factors, and how to create environments so that staff feel confident to escalate their concerns and take action,^98^ are also important elements.

The appropriate audiences for training, and whether training should be focused at the individual level, the team‐level, or the unit level – or all three – needs attention. Critical to progress in this area is a vision of training that takes a whole‐system approach, targeting inter‐professional teamwork, communication, coordination, and ability to mobilise.^99^, ^100^, ^101^, ^102^ There is a nascent evidence base for effective training in maternity care that has established that training for obstetric emergencies is not always effective. Currently, the evidence supports local, multi‐professional training, with integrated teamwork/human factors elements and support tools, for all staff annually^103^ and CTG training should be no different. Indemnifier (NHS Resolution & Victoria Managed Insurance Authority) incentivisation of CTG training follows this evidence base.

In seeking to develop effective training, it must be acknowledged that the evidence‐base is likely to require more purposeful engagement with the ongoing debates and controversies regarding the value and use of CTG.^14^, ^104^, ^105^ For instance, one reason why it may be difficult to demonstrate a positive impact of CTG training on clinical outcomes is the ongoing challenge in determining whether the use of EFM itself improves outcomes. Debates continue about whether the use of CTG truly predicts cerebral palsy and other adverse birth outcomes, and whether its overall effects are positive or negative, given that the rise in caesarean section rates associated with its use has not been accompanied by decreases in rates of obstetric brain injury.^14^ What also remains unclear is whether these challenges are primarily inherent to the technology itself or whether they are primarily implementation and human factor challenges, which could be addressed, in principle, through better training. Improving the evidence‐base for training could therefore help in resolving some of the uncertainties regarding the use of EFM itself.

## Conclusions

Five decades after the introduction of EFM, in a context where many person‐hours are devoted to training each year, and where the consequences for neonatal and maternal outcome are so profound, the findings of this review, suggesting a very poor quality evidence‐base for training, are discomfiting. Although clearly training is essential to quality assurance, better quality studies are required to improve its design, delivery, and evaluation. New research needs not only to deploy better, more robust study designs, but also to re‐imagine what and who training is for – taking proper account of context for monitoring and the team‐based nature of maternity care. Until better data are available, CTG training should be consistent with the evidence base for maternity training: local, multi‐professional with integrated teamworking and support tools.

### Disclosure of interests

MDW, JB, SK, GL, EL, IK, PR and SKi report funding from The Health Foundation to The Healthcare Improvement Studies Institute. TD and CW are members of an RCOG/RCM national fetal monitoring group. Completed disclosure of interests forms are available to view online as supporting information.

### Contribution to authorship

Concept and design were by PR, GL, EL, IK, CW, TD, MDW and JB. Acquisition, analysis or interpretation of data were by SKelly, PR, SKing, COW, GL, EL and JB. The manuscript was drafted by SKelly, PR and JB and it was critically revised for important intellectual content by SKing, COW, GL, EL, IK, CW, TD, MDW and JB. Final approval of the manuscript was given by SKelly, PR, SKing, COW, GL, EL, IK, CW, TD, MDW and JB.

### Details of ethics approval

As this work was a review of published literature in the public domain, ethical approval was not needed.

### Funding

This work was funded by the Health Foundation’s grant to the University of Cambridge for The Healthcare Improvement Studies (THIS) Institute (RG88620). THIS Institute is supported by the Health Foundation – an independent charity committed to bringing about better health and health care for people in the UK. PR is funded by the National Institute for Health Research (NIHR) as an Academic Clinical Lecturer. MDW holds an NIHR Senior Investigator award (NF‐SI‐0617‐10026). The views and opinions expressed by the authors are those of the authors and do not necessarily reflect those of the UK NIHR or the Department of Health and Social Care.

### Acknowledgements

We thank the Cochrane Crowd contributors listed in Appendix S7 for their support of the review through their involvement in additional methodological development work around screening decisions embedded in this review.

## Supporting information

**Table S1.** Studies assessing components and methods for delivery of training (Kirkpatrick level 2 – test results).

**Table S2.** Studies assessing the impact of intrapartum CTG training versus no training on knowledge, behaviours and outcomes.

**Appendix S1.** Search strategies.

**Appendix S2.** Changes from study protocol.

**Appendix S3.** Full data tables with individual study risk of bias assessments Contents

**Appendix S4.** GRADE assessment / summary of findings table Setting: maternity care Contents

**Appendix S5.** Summary risk of bias for individual studies.

**Appendix S6.** Cardiotocography training approaches used in included studies.

**Appendix S7.** Crowd contributors.

Supplementary Material

Supplementary Material

Supplementary Material

Supplementary Material

Supplementary Material

Supplementary Material

Supplementary Material

Supplementary Material

Supplementary Material

Supplementary Material

Supplementary Material

## Data Availability

The data that supports the findings of this study are available in the supplementary material of this article
