# Supplementary material for: Training in the use of intrapartum electronic fetal monitoring with cardiotocography: systematic review and meta‐analysis
Source: BJOG. 2021 Jan 22;128(9):1408–19. doi: 10.1111/1471-0528.16619 (PMC8359372; doi:10.1111/1471-0528.16619)
Supplement: Supplementary file 1 — Table S1. Studies assessing components and methods for delivery of training (Kirkpatrick level 2 – test results). [file BJO-128-1408-s019.pdf]

Table S1. Studies assessing components and methods for delivery of training (Kirkpatrick level 2 – test results)

Study design	Study ID (first author, year)	Intervention (vs comparator) and key results	Outcomes	Effect (sig)	GRADE[1] (overall quality)
**Comparisons of components of training delivery**					
RCT	Evans 1998	Instruction on interventions for women experiencing FHM plus analogy in a written self-instructional lesson; n=41 (vs instruction only; n=47)	Knowledge of concepts, application of rules	nr	Overall quality not assessed[2]
	Kinnick 1990	Comprehensive teaching model (vs teaching model with fewer components); n=68 overall	Test scores for EFM patterns	ns	
	Wilson 2000	Computer assisted learning plus tutorial (vs computer assisted learning alone); n unclear, 51 analysed	Test scores on CTG interpretation	nr	

	Wilson 2001	Computer assisted learning plus tutorial; n unclear, 45 analysed (vs computer assisted learning alone; n unclear, 50 analysed)	Test scores on CTG interpretation	+	
**Comparisons of one method for delivery of training versus another method**					
RCT	Murray 1996	Lecture using transparencies; n=20 (vs computer assisted instruction; n=22)	Test on FHR trace interpretation	ns	Very low
	Wilson 1998	Tutorial; n=5 (vs computer session; n=4)	Test scores on CTG interpretation	ns	
Non-randomised	Keegan 2016	Pre class active learning simulation on a mobile phone; n= 32 allocated, 30 analysed (vs traditional pre-class reading assignment; n=84 allocated, 30 analysed)	10 item quiz on EFM knowledge	+	Very low
	Lee 2019	High fidelity simulation training; n=36 (vs traditional didactic teacher-centred lecture; n=21)	Two standardised written tests (FHR knowledge and interpretation skill) and a	ns (knowl); nr (skill);	

		self-reported questionnaire for perceived clinical management competence.	+ (clin)
O'Boyle 1995	Structured workshop; n=30 (vs learning by videotape; n=30)	Test on ability to interpret EFM strips	ns

Footnotes

[1] - full details of GRADE assessment, including reasons for downgrading, in Appendix S4.

[2] - only risk of bias of individual studies assessed as interventions were too heterogeneous to combine and assess overall quality using GRADE. Individual study risk of bias was High for 3 studies (Kinnick 1990, Wilson 2000, 2001) and Low risk of bias for 1 study (Evans 1998), (Appendix S5).

+ positive effect of CTG training ($p<0.05$) versus no training, or before and after training (i.e. improved outcomes)

ns - no evidence for an effect of training at $p<0.05$ level

nr – statistical relationships not reported (in some cases outcome data was reported but not statistical relationship; or for RCTs in some cases, only within-group comparisons were reported)

n/a – no baseline data for comparison (only follow-up data available)
