# Supplementary material for: Training in the use of intrapartum electronic fetal monitoring with cardiotocography: systematic review and meta‐analysis
Source: BJOG. 2021 Jan 22;128(9):1408–19. doi: 10.1111/1471-0528.16619 (PMC8359372; doi:10.1111/1471-0528.16619)
Supplement: Supplementary file 2 — Table S2. Studies assessing the impact of intrapartum CTG training versus no training on knowledge, behaviours and outcomes. [file BJO-128-1408-s002.pdf]

Table S2. Studies assessing the impact of intrapartum CTG training versus no training on knowledge, behaviours, and outcomes

Outcome	Study design (no. studies)	First author, year	Description of training (vs comparator); number of participants	Outcome assessment	Effect (sig)	GRADE[1] (overall quality)
**Learning as a result of intrapartum CTG training (Kirkpatrick level 2)**						
**Learning assessed through test results**						
	RCT (n=5; 4 in meta-analysis)	Beckley 2000	Computer-assisted teaching (vs no programme); n=117 overall	Multiple choice test on CTG and acid-base balance	+	Low
		Carbonne 2016	E-learning training; n=57 (vs no training; n=56)	Web-based test on CTG	+	
		Devane 2006	Lecture; n=27 (vs non-related video; n=28)	Multiple choice test on FM knowledge and evaluation of tracings.	+	
		Trepannier 1996	1d education programme plus half day review at 6m; n=47 (vs no programme; n=62)	Multiple choice knowledge test	+	
				Clinical skills test, simulated case scenarios.	nr	
		Rizk 2013	Computer-based childbirth simulator and didactic	Test on EFM knowledge	+	

		methods; n=100 (vs routine in-service training; n=100)			
Non-randomised	Cook 2015 (A)	Practical and didactic instruction, 240hrs; n=2	Professional education program testing	nr	Very low
	Cooke 2010	On-line training plus 2 day face to face education; n=954 trained (but n for analysis nr)	Assessment and grading of 2 EFM patterns (normal and pathological)	+	
	Mahley 1999	Didactic presentation and EFM process cards in clinic; n=116 post (n=59 for pre-intervention)	7 multiple choice questions to assess knowledge and interpretation of 1 EFM tracing	+	
	Milde-Luthander 2012	Computer assisted learning programme for interpreting CTG patterns; n=179	Classification of a randomly chosen paper copy of a 40 min long CTG (from 40)	ns	
	Miller 2013	Train the trainer course (1.5d); n=approx. 400 (nr)	Multiple choice test on EFM knowledge	+	
	Stohl 2016	Teaching in FHR monitoring; n=8	Multiple choice and short answer tests on NICHD nomenclature, 3 w after I (6 and 12 m after I also but p value nr)	+	

Thellesen 2017 (Thellesen 2019)	Implementation of a standardised national CTG education programme which consisted of an e-learning program and a 1-day course; n=790	30 item CTG multiple choice test	nr
Daglar 2019	Theoretical and practical training on trace interpretation through individual and group studies to final year midwifery students; n=103	Test on EFM knowledge and interpretation of 10 traces.	+
Froc 2018	Theoretical and practical training on trace interpretation conducted by a pair of expert midwives-obstetricians, followed by a theoretical reminder of EFM basic rules such as communication, asking for	Mean test scores assessed using a 10-item questionnaire.	+

	advice, cross-monitoring); n=234		
Jomeen 2019	Face-to-face lecture-based day aimed at providing evidence-based training on CTG interpretation; n=255	Knowledge assessed using a 10 item true-false questionnaire	+
Parsons 2013	One-to-one education with 3 live tracings; n=25	Individual performance for the interpretation and management of three CTG traces rated on a 0-4 Likert scale.	+
Di Lieto 2002	Theoretical and practical training in telemedicine system, including computerised analysis of CTG traces, guidelines for CTG traces and interpretation and diagnostic-therapeutic protocols; n=nr	Evaluation questionnaire including one CTG trace and 5 multiple choice questions.	+
Pettker 2011	EFM training and certification, as part of a	Success rate at the EFM certification test	+

			broader patient safety programme; n=nr.			
	Quantitative descriptive	Rehling-Anthony 2011(A)	Implementation of a standardised education and competence validation programme; n=105	Exam to obtain EFM certification	n/a	
		Guild 1994	Fetal monitoring computer tutorial; n=nr	Assessment of 6 CTG strips and 25-item multiple choice test.	n/a	
**Learning assessed through inter-observer agreement of CTG interpretations (Kirkpatrick level 2)**						
	Non-randomised	Ayres-de-Campos	Training of clinicians in author-specific FHR diagnostic criteria; n=3 (vs usual practice; n=3)	FHR baseline estimation of 300 tracings assessed using 3 observers (between group values nr)	nr	Very low
		Davis 2010 (A)	Auditing with feedback to identify learning needs in nurses; n=nr	Agreement between auditors and nurses in FHM interpretation (monthly 40 charts, 20 labour inductions, 20 Pitocin augmentations)	nr	
		Govindappagari 2016	Pre-post – mandatory 9h online course in EFM management and interpretation; n=351	Agreement between obstetric care providers (e.g. physician assistants, residents) and nurses	+	

Quantitative descriptive	Blix 2005	6h CTG training programme for midwives and obstetricians; n=6	Agreement between pairs of observers for classification of CTG (normal/intermediary/abnormal)	nr	

Learning assessed through performance in simulated scenarios (Kirkpatrick level 2)

RCTs	Rizk 2013	Computer-based childbirth simulator and didactic methods; n=100 (vs routine in-service training; n=100)	Proportion of nurses with satisfactory in-hospital CTG performance score assessed using a checklist.	+	Low
	Cuerva 2018	Simulation-based training including CTG training with demonstration of a properly performed childbirth scenario n=12 (vs simulation training without demonstration of properly performed scenario; n=12)	Evaluation of performance assessed by teachers using a 5 point scale (separate CTG evaluation)	ns	
	Fransen 2013 (cluster RCT)	1 day training session including CTG training in a medical simulation centre;	Teamwork performance assessed by independent experts using the Clinical	+	

			n=12 hospitals (vs no training; n=12 hospitals)	Teamwork Scale (CTS) 6 mo after training in a clinically simulated scenario.		
**Behaviours following CTG training (Kirkpatrick level 3)**						
	Non-randomised	Cook 2015 (A)	CTG training involving 240 hrs of both didactic and practical instruction; n=2	Unclear – study authors reported that nurses self-reported confidence improved.	nr	Very low
		Davis 2010 (A)	CTG training involving regular auditing and identifying learning needs; n=nr	Unclear – study authors reported improved awareness of patient safety and collegial discussions of FHM tracings.	nr	
		Pettker 2009	Educational programme as part of a wider organisational strategic improvement for patient safety; n=nr	Ratings for 'good teamwork' across all staff assessed by Safety Attitudes Questionnaire.	nr	
		Vadnais 2011	Multifaceted education including pocket cards, posters, didactic sessions, online educational programme; n=nr	Compliance assessed via random chart review	+	

Wagner 2012	Mandatory EFM educational course and exam, team training with focus on documentation and appropriate management of FHR abnormalities; n=nr; but approx. 1948 completed survey.	Documentation of fetal heart rate abnormalities, appropriate management of FHR abnormalities, staff perceptions of patient safety, assessed using survey	+
Gnanasambanthan 2018 (A)	Structured education programme for midwives and doctors including mandatory CTG masterclasses, weekly teaching with trained midwives and obstetricians; n=nr, n=36 patient's charts audited.	Documentation of CTG interpretation; % 'fresh-eyes' checking of EFM interpretation by a second healthcare professional; % implementation of action plan where CTG trace was suspicious or pathological.	nr
Grace 2018 (A)	Interactive training in fetal heart rate monitoring,	"confidence in ability and understanding in most subject areas". "improvements in safe functioning on lab/delivery unit,	nr

			labour management and teamwork; n=nr	interpretation of EFM and intervention, use of communication tools and participation in emergency CS.		
		Jomeen 2019	Face-to-face lecture-based day aimed at providing evidence-based training on CTG interpretation; n=255.	Confidence assessed using a 10 item true-false questionnaire and attitudes assessed using open-ended qual questions	+	
	Quantitative descriptive	Burke 2013	Mandatory 2 h didactic and simulation session covering multiple obstetric issues with one element being fetal monitoring; n=372.	Perceptions of safety assessed using AHRQ safety questionnaire	+	
**Maternal/fetal outcomes following CTG training (Kirkpatrick level 4)**						
Hypoxic ischaemic encephalopathy (HIE)	Non-randomised	Brown 2017	EFM one day course plus online and hard copy resource materials	HIE<br>RR 0.82 (95% CI 0.76, 0.89)<br>Total births: n =1606218 (before); 1906488 (after)	Lower risk	Low
		Draycott 2006	1d training course on CTG interpretation with	HIE overall<br>RR 0.50 (95% CI 0.26, 0.95)	Lower risk	

		obstetric emergency drill stations.	Total births: n= 8430 (before); 11030 (after)	
		As above	Moderate/severe HIE<br>RR 0.53 (95% CI 0.24, 1.13)	ns
	Byford 2014	Fetal surveillance educational programme, practical guide, online education programme.	HIE<br>Sig lower p=0.02 (graphical data)<br>Total births: n=156558 (before); 364612 (after)	Lower risk
	Chandraharan 2014 (A)	"Intensive fetal training" and a mandatory test in fetal monitoring.	HIE<br>1.2/ 1000 (before); 1.1/1000 after, p=ns<br>Total births: n=5167 (before); 3804 (after)	ns
	Wijemanne 2016 (A)	Intensive physiology-based CTG training and mandatory competency testing	HIE (no pre-post comparison reported)	nr
Quantitative descriptive	Al-Samarrai 2019 (A)	Physiologically based CTG training and supported advanced assessment and decision making plus human factors training since 2016; n=nr	HIE (1-3 rate) (nr)	nr

Rates of emergency caesarean section	Non-randomised	Brown 2017	EFM one day course plus online and hard copy resource materials.	Emergency caesarean section rates<br>RR 0.95 (95% CI 0.95, 0.96)<br>Total births (as above)	Lower rate	Very low
		Draycott 2006	1d training course on CTG interpretation with obstetric emergency drill stations.	Emergency caesarean section rates<br>784/8430 (9.3%) (before); 1254/11030 (11.4%) after; p<0.001	Higher rate	
		Chandraharan 2014 (A)	"Intensive fetal training" and a mandatory test in fetal monitoring.	Emergency caesarean section rates<br>775/5167 (15%) (before); 342/3804 (9%) (after); p<0.0001	Lower rate	
		Thellesen 2019 (Thellesen 2017)	Implementation of a standardised national CTG education programme: e-learning program and a 1-day course; n= 2094 trained;	Emergency caesarean rates<br>At impl: RR 1.05 (1.01, 1.08); 3 m post impl RR 0.98 (0.96, 1.01)<br>Total births: n=331282	Higher rate (impl); ns (post 3 months)	
Proportion of babies with low Apgar (<5; </=6; <7) at 5 minutes	Non-randomised	Brown 2017	EFM one day course plus online and hard copy resource materials.	Proportion of babies with APGAR <5 at 5 min<br>RR 0.82 (95% CI 0.7, 0.87)	Lower risk	Low
		Draycott 2006	1d training course on CTG interpretation with	Proportion of babies with APGAR </=6 at 5 mins	Lower risk	

			obstetric emergency drill stations.	RR 0.51 (95% CI 0.35, 0.74)		
		Ting 2017	1h session with lecture, case base discussion, and video based on situation-background-assessment-recommendation technique (SBAR).	Proportion of babies with APGAR <7 at 5 mins<br>Pre 145/3368 (4.3%); 60/1346 (4.5%); p nr	nr	
		Thellesen 2019 (Thellesen 2017)	Implementation of a standardised national CTG education programme: e-learning program and a 1-day course; n= 2094 trained; outcomes based on n=331282 live singleton births.	OR babies with APGAR <7 at 5 mins<br>At impl: RR 0.97 (0.84, 1.11); 3 m post impl RR 0.99 (0.90, 1.10)<br>Total births: n=331282	ns	
Neonatal deaths	Non-randomised	Brown 2017	EFM one day course plus online and hard copy resource materials.	Overall neonatal deaths<br>RR 1.04 (95% CI 0.88, 1.23)	ns	Very low
			As above	Hypoxic intrapartum perinatal deaths<br>RR 0.49 (95% CI 0.35, 0.68)	Lower risk	

	As above	Neonatal mortality amongst babies admitted to neonatal unit<br>RR 0.76 (95% CI 0.65, 0.89)	Lower risk
Chandraharahan 2014 (A)	"Intensive fetal training" and a mandatory test in fetal monitoring.	Overall neonatal death rates<br>Pre 1.7/1000; post 1.3/1000 p=ns (value nr)	ns

Footnotes:

[1] – full details of GRADE assessment, including reasons for downgrading, in Appendix S4. GRADE is a summary of the overall quality of the body of evidence (for several studies), not the individual study. Full details of individual study assessments are in Appendices S3 and S5.

[2] - the terminology used is as described in the study i.e. CTG (cardiotocography), EFM (electronic fetal monitoring), FHM (fetal heartrate monitoring), FM (fetal monitoring) are all used to describe electronic fetal monitoring with cardiotocography.

(A) Abstract only available

I = intervention

RR=relative risk

95% CI=95% confidence interval

+ - positive effect of CTG training (p<0.05) versus no training, or before and after training (i.e. lower risk or improved outcomes)

ns - no evidence for an effect of training at p<0.05 level (or between group effect for the appropriate comparison)

nr – statistical relationships not reported (in some cases outcome data was reported but not statistical relationship)

n/a – no baseline data for comparison (only follow-up data available)
