# Supplementary material for: Training in the use of intrapartum electronic fetal monitoring with cardiotocography: systematic review and meta‐analysis
Source: BJOG. 2021 Jan 22;128(9):1408–19. doi: 10.1111/1471-0528.16619 (PMC8359372; doi:10.1111/1471-0528.16619)
Supplement: Supplementary file 3 — Appendix S1. Search strategies. [file BJO-128-1408-s006.pdf]

**Appendix S1.** Search strategies

Contents

1 Database searches ........ 2

1.1 Search overview ........ 2

1.2 Search strategies ........ 2

2 Grey Literature searches ........ 11

2.1 Search overview ........ 11

2.2 Search strategies ........ 11

3 Ongoing clinical trials register searches ........ 12

## 1 Database searches

### 1.1 Search overview

Searches were originally conducted between 18 and 20 December 2017 and updated on 24 July 2019. And updated till end October 2019.

| Table A1. Summary of database searches | | | |
|---|---|---|---|
| Databases | Total hits at Dec 2017 | No. of additional hits by July 2019 | No. of additional hits by end Oct 2019 |
| Medline via OVID | 1549 | 155 | 41 |
| Embase via OVID | 2685 | 288 | 52 |
| Pubmed | 2295 | 260 | 48 |
| CINAHL via Ebsco | 506 | 62 | 22 |
| PsycINFO via Ebsco | 131 | 3 | 0 |
| Web of Science Core Collection | 5499 | 990 | 430 |
| Scopus | 5037 | 492 | 205 |
| Cochrane – Central Register of Controlled Trials | 7 | 0 | 0 |
| British Nursing Index via ProQuest | 336 | 24 | 6 |
| ERIC via Ebsco | 23 | 6 | 0 |
| PQDT via ProQuest | 124 | 7 | 8 |
| Total | | 2287 | 806 |
| Deduplicated | 13856 | 1606 (against previous) | 612 (against previous) |

### 1.2 Search strategies

#### 1.2.1 Medline via Ovid

Original search

1 (course* or syllabus* or student* or teach or lesson* or feedback or classroom* or tutorial* or tutor* or seminar* or presentation* or practical* or online study* or fieldwork* or "face to face" or "online learning*" or "e learning*" or demonstration* or "computer based*" or "case studies" or "case study").ti,ab. or Clinical Competence/ or training*.ti,ab. or "clinical competenc*".ti,ab. or "clinical skill*".ti,ab. or Education/ or workshop*.ti,ab. or "training program*".ti,ab. or "educational activit*".ti,ab. or "competency based education".ti,ab. or Competency-Based Education/

2 Cardiotocography/ or "fetal monitor*".ti,ab. or "foetal monitor*".ti,ab. or CTG.ti,ab. or cardiotoco*.ti,ab. or "foetal heartbeat*".ti,ab. or "fetal heartbeat*".ti,ab. or "foetal distress".ti,ab. or "fetal distress".ti,ab. or "foetal distress".ti,ab. or "fetal distress".ti,ab. or "late deceleration*".ti,ab. or "early deceleration*".ti,ab. or "foetal heart acceleration*".ti,ab. or "fetal heartrate".ti,ab. or "foetal heart rate".ti,ab. or "continuous electronic monitoring in labour".ti,ab. or "continuous electronic monitoring intrapartum".ti,ab. or "continuous electronic monitoring in delivery".ti,ab. or "foetal heart variability*".ti,ab.

3 Animal/ not Human/

4 (1 and 2) not 3

Update search (additional terms) July 2019

5 ((201712* or 2018* or 2019* or 2020*).dp. or (201712* or 2018* or 2019* or 2020*).ez. or (201712* or 2018* or 2019* or 2020*).ed.)
6 4 and 5

Updated search (additional terms) Oct 2019

5 ((201907* or 201908* or 201909* or 201910*).dp. or (201907* or 201908* or 201909* or 201910*).ez. or (201907* or 201908* or 201909* or 201910*).ed.)

### 1.2.2 Embase via Ovid

Original search

((course*.ti,ab OR syllabus*.ti,ab OR student*.ti,ab OR teach.ti,ab OR lesson*.ti,ab OR feedback.ti,ab OR classroom*.ti,ab OR tutorial*.ti,ab OR tutor*.ti,ab OR seminar*.ti,ab OR presentation*.ti,ab OR practical*.ti,ab OR online study*.ti,ab OR fieldwork*.ti,ab OR "face to face".ti,ab OR "online learning*".ti,ab OR "e learning*".ti,ab OR demonstration*.ti,ab OR "computer based*".ti,ab OR "case studies".ti,ab OR "case study".ti,ab OR Clinical Competence/ OR "clinical competenc*".ti,ab OR "clinical skill*".ti,ab OR training*.ti,ab OR Education/ OR "workshop*".ti,ab OR "training program*".ti,ab OR "educational activit*".ti,ab OR "competency based education".ti,ab OR Competency-Based Education/) AND (Cardiotocography/ OR "fetal monitor*".ti,ab OR "foetal monitor*".ti,ab OR CTG.ti,ab OR cardiotoco*.ti,ab OR "foetal heartbeat*".ti,ab OR "fetal heartbeat*".ti,ab OR "foetal distress".ti,ab OR "fetal distress".ti,ab OR "foetal distress".ti,ab OR "fetal distress".ti,ab OR "late deceleration*".ti,ab OR "early deceleration*".ti,ab OR "foetal heart acceleration*".ti,ab OR "fetal heartrate".ti,ab OR "foetal heart rate".ti,ab OR "continuous electronic monitoring in labour".ti,ab OR "continuous electronic monitoring intrapartum".ti,ab OR "continuous electronic monitoring in delivery".ti,ab OR "foetal heart variability*".ti,ab OR "foetal heart variability*".ti,ab)) NOT ((exp animal/ or nonhuman/) NOT exp human/)

Update search (additional terms) July 2019

AND

((201712* or 2018* or 2019* or 2020*).dc. or (201712* or 2018* or 2019* or 2020*).dd)

Update search (additional terms) Oct 2019

AND

((201907* or 201908* or 201909* or 201910*).dc. or (201907* or 201908* or 201909* or 201910*).dd)

### 1.2.3 PubMed

Original search: 20/12/17 (1889);
Update search (July 2019): Filter 1/12/2017 to 31/12/2020
Update search (Oct 2019) (("2019/07/01"[Date - Entry] : "2019/10/31"[Date - Entry]))

((((((((((((((((((((((((((course*[Title/Abstract]) OR syllabus*[Title/Abstract]) OR student*[Title/Abstract]) OR teach*[Title/Abstract]) OR lesson*[Title/Abstract]) OR feedback*[Title/Abstract]) OR classroom*[Title/Abstract]) OR tutorial*[Title/Abstract]) OR tutor*[Title/Abstract]) OR seminar*[Title/Abstract]) OR presentation*[Title/Abstract]) OR practical*[Title/Abstract]) OR online study*[Title/Abstract]) OR fieldwork*[Title/Abstract]) OR face to face[Title/Abstract]) OR online learning*[Title/Abstract]) OR e learning*[Title/Abstract]) OR demonstration*[Title/Abstract]) OR computer based*[Title/Abstract]) OR case studies[Title/Abstract]) OR case study*[Title/Abstract])) OR (((clinical competence[MeSH Terms]) OR clinical competenc*[Title/Abstract]) OR clinical skill*[Title/Abstract])) OR ((((education[MeSH Terms]) OR workshop*[Title/Abstract]) OR training*[Title/Abstract] OR training program*[Title/Abstract]) OR educational activit*[Title/Abstract])) OR ((competency based education[Title/Abstract]) OR competency based education[MeSH Terms]))) AND (((((((Cardiotocography[MeSH Terms]) OR fetal monitor*[Title/Abstract]) OR foetal monitor*[Title/Abstract]) OR CTG[Title/Abstract]) OR cardiotoco*[Title/Abstract] OR foetal heartbeat*[Title/Abstract] OR fetal heartbeat[Title/Abstract] OR foetal distress[Title/Abstract] OR fetal distress[Title/Abstract] OR foetal distress[Title/Abstract] OR fetal distress[Title/Abstract] OR late deceleration*[Title/Abstract] OR fetal heartrate[Title/Abstract]) OR foetal heart rate[Title/Abstract]) OR continuous electronic monitoring in labour[Title/Abstract]) OR continuous electronic monitoring intrapartum[Title/Abstract]) OR continuous electronic monitoring in delivery[Title/Abstract] OR early deceleration*[Title/Abstract] OR foetal heart acceleration*[Title/Abstract] OR foetal heart variability*[Title/Abstract])))) NOT ((animals [mh] NOT humans [mh])))

### 1.2.4 1.2.4 Cochrane Central

ID Search Hits

#1 course*.ti,ab. OR syllabus*.ti,ab. OR student*.ti,ab. OR teach.ti,ab. OR lesson*.ti,ab. OR feedback.ti,ab. OR classroom*.ti,ab. OR tutorial*.ti,ab. OR tutor*.ti,ab. OR seminar*.ti,ab. OR presentation*.ti,ab. OR practical*.ti,ab. OR online study*.ti,ab. OR fieldwork*.ti,ab. OR "face to face".ti,ab. OR "online learning*".ti,ab. OR "e learning*".ti,ab. OR demonstration*.ti,ab. OR "computer based*".ti,ab. OR "case studies".ti,ab. OR "case study".ti,ab. OR "clinical competenc*".ti,ab. OR training*.ti,ab. OR "clinical skill*".ti,ab. OR workshop*.ti,ab. OR "training program*".ti,ab. OR "educational activit*".ti,ab. OR "competency based education".ti,ab. 23962

#2 MeSH descriptor: [Clinical Competence] explode all trees 3033

#3 MeSH descriptor: [Education] explode all trees 30274

#4 MeSH descriptor: [Competency-Based Education] explode all trees 81

#5 #1 or #2 or #3 or #4 51635

#6 MeSH descriptor: [Cardiotocography] explode all trees 140

#7 "fetal monitor*".ti,ab. OR "foetal monitor*".ti,ab. OR CTG.ti,ab. OR cardiotoco*.ti,ab. OR "foetal heartbeat*".ti,ab. OR "fetal heartbeat*".ti,ab. OR "foetal distress".ti,ab. OR "fetal distress".ti,ab. OR "foetal distress".ti,ab. OR "fetal distress".ti,ab. OR "late deceleration*".ti,ab. OR "early deceleration*".ti,ab. OR "foetal heart acceleration*".ti,ab. OR "fetal heartrate".ti,ab OR "foetal heart rate".ti,ab OR "continuous electronic monitoring in

labour”.ti,ab OR “continuous electronic monitoring intrapartum”.ti,ab OR “continuous electronic monitoring in delivery”.ti,ab OR “foetal heart variability*”.ti,ab OR “foetal heart variability*”.ti,ab 5556

| | | |
|---|---|---|
| #8 | #6 or #7 | 5624 |
| #9 | #5 and #6 | 7 |

Updated search (additional terms)

With Publication Year from 2019 to 2019, with Cochrane Library publication date from Jul 2019 to Oct 2019, in Trials

### 1.2.5 PsycINFO via Ebscohost

Original search

(TI course* OR TI syllabus* OR TI student* OR TI teach* OR TI lesson* OR TI feedback OR TI classroom* OR TI tutorial* OR TI tutor* OR TI seminar* OR TI presentation* OR TI practical* OR TI (online study*) OR TI fieldwork* OR TI (face to face) OR TI (online learning*) OR TI (e learning*) OR TI demonstration* OR TI (computer based*) OR TI (case studies) OR TI (case study) OR TI (clinical competenc*) OR TI (clinical skill*) OR TI workshop* OR TI (training program*) OR TI (educational activit*) OR TI (competency based education) OR AB course* OR AB syllabus* OR AB student* OR AB teach* OR AB lesson* OR AB feedback OR AB classroom* OR AB tutorial* OR AB tutor* OR AB seminar* OR AB presentation* OR AB practical* OR AB (online study*) OR AB fieldwork* OR AB (face to face) OR AB (online learning*) OR AB (e learning*) OR TI training* OR AB training* OR AB demonstration* OR AB (computer based*) OR AB (case studies) OR AB (case study”) OR AB (clinical competenc*) OR AB (clinical skill*) OR AB workshop* OR AB (training program*) OR AB (educational activit*) OR AB (competency based education) OR DE “Education”)

AND

(TI (fetal monitor*) OR TI (foetal monitor*) OR TI CTG OR TI cardiotoco* OR TI (foetal heartbeat*) OR TI (fetal heartbeat*) OR TI (foetal distress) OR TI (fetal distress) OR TI (foetal distress) OR TI (late deceleration*) OR TI (early deceleration*) OR TI (foetal heart acceleration*) OR TI (foetal heart variability*) OR AB (fetal monitor*) OR AB (foetal monitor*) OR AB CTG OR AB cardiotoco* OR AB (foetal heartbeat*) OR AB (fetal heartbeat*) OR AB (foetal distress) OR AB (fetal distress) OR AB (foetal distress) OR AB (late deceleration*) OR AB (early deceleration*) OR AB (foetal heart acceleration*) OR AB (fetal heartrate) OR AB (foetal heart rate) OR AB (continuous electronic monitoring in labour) OR AB (continuous electronic monitoring intrapartum) OR AB (continuous electronic monitoring in delivery) OR TI (fetal heartrate) OR TI (foetal heart rate) OR TI (continuous electronic monitoring in labour) OR TI (continuous electronic monitoring intrapartum) OR TI (continuous electronic monitoring in delivery) OR AB (foetal heart variability*))

Update search (additional terms) July 2019

AND

((EM 201712  or DT 2020 or DT 2019 or DT 2018 )

Update search (additional terms) Oct 2019

AND

Limiters - Published Date: 20190701-20191031

### 1.2.6 CINAHL via Ebscohost

Original search

TI course* OR TI syllabus* OR TI student* OR TI teach* OR TI lesson* OR TI feedback OR TI classroom* OR TI tutorial* OR TI tutor* OR TI seminar* OR TI presentation* OR TI practical* OR TI (online study*) OR TI fieldwork* OR TI (face to face) OR TI (online learning*) OR TI (e learning*) OR TI demonstration* OR TI (computer based*) OR TI (case studies) OR TI (case study) OR TI (clinical competenc*) OR TI (clinical skill*) OR TI workshop* OR TI (training program*) OR TI (educational activit*) OR TI (competency based education) OR AB course* OR AB syllabus* OR AB student* OR AB teach* OR AB lesson* OR AB feedback OR AB classroom* OR AB tutorial* OR AB tutor* OR AB seminar* OR AB presentation* OR AB practical* OR AB (online study*) OR AB fieldwork* OR AB (face to face) OR AB (online learning*) OR AB (e learning*) OR AB demonstration* OR AB (computer based*) OR AB (case studies) OR AB (case study) OR AB (clinical competenc*) OR AB (clinical skill*) OR AB workshop* OR AB (training program*) OR TI training* OR AB training* OR AB (educational activit*) OR AB (competency based education) OR MH “Clinical Competence” OR MH “Education” OR MH “Education, Competency-Based”

AND

TI (fetal monitor*) OR TI (foetal monitor*) OR TI CTG OR TI cardiotoco* OR TI (foetal heartbeat*) OR TI (fetal heartbeat*) OR TI (foetal distress) OR TI (fetal distress) OR TI (foetal distress) OR TI (late deceleration*) OR TI (early deceleration*) OR TI (foetal heart acceleration*) OR TI (foetal heart variability*) OR AB (fetal monitor*) OR AB (foetal monitor*) OR AB CTG OR AB cardiotoco* OR AB (foetal heartbeat*) OR AB (fetal heartbeat*) OR AB (foetal distress) OR AB (fetal distress) OR AB (foetal distress) OR AB (late deceleration*) OR AB (early deceleration*) OR AB (foetal heart acceleration*) OR AB (fetal heartrate) OR AB (foetal heart rate) OR AB (continuous electronic monitoring in labour) OR AB (continuous electronic monitoring intrapartum) OR AB (continuous electronic monitoring in delivery) OR TI (fetal heartrate) OR TI (foetal heart rate) OR TI (continuous electronic monitoring in labour) OR TI (continuous electronic monitoring intrapartum) OR TI (continuous electronic monitoring in delivery) OR AB (foetal heart variability*) OR MH “Cardiotocography”

NOT (MH “Animals” NOT MH “Human”)

Update search (additional terms) July 2019

AND

((EM 201712  or DT 2020 or DT 2019 or DT 2018 )

Update search (additional terms) Oct 2019

AND

Limiters - Published Date: 20190701-20191031

### 1.2.7 British Nursing Index via Proquest

Original search

((ti,ab(course*) OR ti,ab(syllabus*) OR ti,ab(student*) OR ti,ab(teach*) OR ti,ab(lesson*) OR ti,ab(feedback) OR ti,ab(classroom*) OR ti,ab(tutorial*) OR ti,ab(tutor*) OR ti,ab(seminar*) OR ti,ab(presentation*) OR ti,ab(practical*) OR ti,ab(online study*) OR ti,ab(fieldwork*) OR ti,ab(face to face) OR ti,ab(online learning*) OR ti,ab(e learning*) OR ti,ab(demonstration*) OR ti,ab(computer based*) OR ti,ab(case studies) OR ti,ab(case study) OR ti,ab(clinical competenc*) OR ti,ab(clinical skill*) OR ti,ab(training*) OR ti,ab(workshop*) OR ti,ab(training program*) OR ti,ab(educational activit*) OR ti,ab(competency based education) OR mesh.Exact("Education") OR mesh.Exact("Clinical Competence")) AND (ti,ab(fetal monitor*) OR ti,ab(foetal monitor*) OR ti,ab(cog) OR ti,ab(cardiotoco*) OR ti,ab(foetal heartbeat*) OR ti,ab(fetal heartbeat*) OR ti,ab(foetal distress) OR ti,ab(fetal distress) OR ti,ab(foetal distress) OR ti,ab(late deceleration*) OR ti,ab(early deceleration*) OR ti,ab(foetal heart acceleration*) OR ti,ab(fetal heartrate) OR ti,ab(foetal heart rate) OR ti,ab(continuous electronic monitoring in labour) OR ti,ab(continuous electronic monitoring intrapartum) OR ti,ab(continuous electronic monitoring in delivery) OR ti,ab(foetal heart variability*) OR ti,ab(fetal monitor*) OR mesh.Exact("Cardiotocography"))) NOT (mesh.Exact("Animals") NOT mesh.Exact("Humans"))

Update search (July 2019)

August 2017 to June 2019

Update search (additional terms) Oct 2019

- Additional limits -Date: From July 2019 to October 2019

### 1.2.8 ERIC via Ebscohost

Original search

(TI course* OR TI syllabus* OR TI student* OR TI teach* OR TI lesson* OR TI feedback OR TI classroom* OR TI tutorial* OR TI tutor* OR TI seminar* OR TI presentation* OR TI practical* OR TI (online study*) OR TI fieldwork* OR TI (face to face) OR TI (online learning*) OR TI (e learning*) OR TI demonstration* OR TI (computer based*) OR TI (case studies) OR TI (case study) OR TI (clinical competenc*) OR TI (clinical skill*) OR TI workshop* OR TI (training program*) OR TI (educational activit*) OR TI (competency based education) OR TI training* OR AB training* OR AB course* OR AB syllabus* OR AB student* OR AB teach* OR AB lesson* OR AB feedback OR AB classroom* OR AB tutorial* OR AB tutor* OR AB seminar* OR AB presentation* OR AB practical* OR AB (online study*) OR AB fieldwork* OR AB (face to face) OR AB (online learning*) OR AB (e learning*) OR AB demonstration* OR AB (computer based*) OR AB (case studies) OR AB (case study) OR AB

(clinical competenc*) OR AB (clinical skill*) OR AB workshop* OR AB (training program*) OR AB (educational activit*) OR AB (competency based education) OR Education) AND (TI (fetal monitor*) OR TI (foetal monitor*) OR TI CTG OR TI cardiotoco* OR TI (foetal heartbeat*) OR TI (fetal heartbeat*) OR TI (foetal distress) OR TI (fetal distress) OR TI (foetal distress) OR TI (late deceleration*) OR TI (early deceleration*) OR TI (foetal heart acceleration*) OR TI (foetal heart variability*) OR AB (fetal monitor*) OR AB (foetal monitor*) OR AB CTG OR AB cardiotoco* OR AB (foetal heartbeat*) OR AB (fetal heartbeat*) OR AB (foetal distress) OR AB (fetal distress) OR AB (foetal distress) OR AB (fetal heartrate) OR AB (foetal heart rate) OR AB (continuous electronic monitoring in labour) OR AB (continuous electronic monitoring intrapartum) OR AB (continuous electronic monitoring in delivery) OR TI (fetal heartrate) OR TI (foetal heart rate) OR TI (continuous electronic monitoring in labour) OR TI (continuous electronic monitoring intrapartum) OR TI (continuous electronic monitoring in delivery) OR AB (late deceleration*) OR AB (early deceleration*) OR AB (foetal heart acceleration*) OR AB (foetal heart variability*))

Limiters - Date Published: 20170901-20201231

Update search (July 2019)

Limiters - Date Published: 20170901-20201231

Update search (Oct 2019)

Limiters - Date Published: 20190701-20191031

### 1.2.9 Scopus

Original search

( ( TITLE-ABS-KEY ( fetal W/1 monitor* ) OR TITLE-ABS-KEY ( foetal w/1 monitor* ) OR TITLE-ABS-KEY ( ctg ) OR TITLE-ABS-KEY ( cardiotoco* ) OR TITLE-ABS-KEY ( foetal w/1 heartbeat* ) OR TITLE-ABS-KEY ( fetal w/1heartbeat* ) OR TITLE-ABS-KEY ( foetal w/1 distress ) OR TITLE-ABS-KEY ( fetal w/1 distress ) OR TITLE-ABS-KEY ( foetal w/1 distress ) OR TITLE-ABS-KEY ( late w/1 deceleration* ) OR TITLE-ABS-KEY ( early w/1 deceleration* ) OR TITLE-ABS-KEY ( foetal w/1 heart w/1 acceleration* ) OR TITLE-ABS-KEY ( foetal w/1 heart w/1 variability* ) OR TITLE-ABS-KEY ( fetal w/1 heartrate ) OR TITLE-ABS-KEY ( foetal w/1 heart w/1 rate ) OR TITLE-ABS-KEY ( continuous w/1 electronic W/1 monitoring W/1 in W/1 labour ) OR TITLE-ABS-KEY ( continuous W/1 electronic W/1 monitoring W/1 intrapartum ) OR TITLE-ABS-KEY ( continuous W/1 electronic W/1 monitoring W/1 in W/1 delivery ) OR TITLE-ABS-KEY ( fetal W/1 heartrate ) OR TITLE-ABS-KEY ( foetal W/1 heart W/1 rate ) OR TITLE-ABS-KEY ( continuous W/1 electronic W/1 monitoring W/1 in W/1 labour ) OR TITLE-ABS-KEY ( continuous W/1 electronic W/1 monitoring W/1 intrapartum ) OR TITLE-ABS-KEY ( continuous W/1 electronic W/1 monitoring W/1 in W/1 delivery ) OR TITLE-ABS-KEY ( fetal W/1 monitor* ) OR TITLE-ABS-KEY ( cardiotocography ) ) AND ( TITLE-ABS-KEY ( course* ) OR TITLE-ABS-KEY ( syllabus* ) OR TITLE-ABS-KEY ( student* ) OR TITLE-ABS-KEY ( teach* ) OR TITLE-ABS-KEY ( lesson* ) OR TITLE-ABS-KEY ( feedback ) OR TITLE-ABS-KEY ( classroom* ) OR TITLE-ABS-KEY ( tutorial* ) OR TITLE-ABS-KEY ( tutor* ) OR TITLE-ABS-KEY ( seminar* ) OR TITLE-ABS-KEY ( presentation* ) OR TITLE-ABS-KEY ( practical* ) OR TITLE-ABS-KEY ( online W/1 study* ) OR TITLE-ABS-KEY ( fieldwork* ) OR TITLE-ABS-KEY ( face W/1 to W/1 face ) OR TITLE-ABS-KEY ( online W/1 learning* ) OR TITLE-ABS-KEY ( e-learning* ) OR TITLE-ABS-KEY ( demonstration* ) OR TITLE-ABS-KEY (

computer W/1 based* ) OR TITLE-ABS-KEY ( case W/1 studies ) OR TITLE-ABS-KEY ( case W/1 study ) OR TITLE-ABS-KEY ( clinical W/1 competenc* ) OR TITLE-ABS-KEY ( clinical W/1 skill* ) OR TITLE-ABS-KEY ( workshop* ) OR TITLE-ABS-KEY ( training W/1 program* ) OR TITLE-ABS-KEY ( educational W/1 activit* ) OR TITLE-ABS-KEY ( training* ) OR TITLE-ABS-KEY ( competency W/1 based W/1 education ) OR TITLE-ABS-KEY ( education ) OR TITLE-ABS-KEY ( clinical W/1 competence ) ) ) AND NOT ( TITLE-ABS-KEY ( animal* ) not TITLE-ABS-KEY ( humans* ) )

Note: Scopus does not have MeSH terms, so the TITLE-ABS-KEY search string is the closest replication of a title, abstract and MeSH search.

Update search

July 2019: Limiters - Date Published: 20170901-20191231

Oct 2019: Limiters - Date Published: 20190101-20201231

1.2.10 Web of Science (limited to the Web of Science Core Collection)

Original search

TS=(course*) OR TS=(syllabus*) OR TS=(student*) OR TS=(teach*) OR TS=(lesson*) OR TS=(feedback) OR TS=(classroom*) OR TS=(tutorial*) OR TS=(tutor*) OR TS=(seminar*) OR TS=(presentation*) OR TS=(practical*) OR TS=(online study*) OR TS=(fieldwork*) OR TS=(face to face) OR TS=(online learning*) OR TS=(e learning*) OR TS=(demonstration*) OR TS=(computer based*) OR TS=(case studies) OR TS=(case study) OR TS=(clinical competenc*) OR TS=(clinical skill*) OR TS=(workshop*) OR TS=(training program*) OR TS=(educational activit*) OR TS=(competency based education) OR TS=(training*) OR TS=(education) OR TS=(clinical competence)

AND

TS=(fetal monitor*) OR TS=(foetal monitor*) OR TS=(CTG) OR TS=(cardiotoco*) OR TS=(foetal heartbeat*) OR TS=(fetal heartbeat*) OR TS=(foetal distress) OR TS=(fetal distress) OR TS=(foetal distress) OR TS=(late deceleration*) OR TS=(early deceleration*) OR TS=(foetal heart acceleration*) OR TS=(fetal heartrate) OR TS=(foetal heart rate) OR TS=(continuous electronic monitoring in labour) OR TS=(continuous electronic monitoring intrapartum) OR TS=(continuous electronic monitoring in delivery) OR TS=(foetal heart variability*) OR TS=(fetal monitor*) OR TS=(cardiotocography)

NOT (TS=(animal*) NOT TS=(humans*))

Update search

July 2019: Refined by: PUBLICATION YEARS: ( 2019 OR 2018 OR 2017 )

Oct 2019: Refined by: PUBLICATION YEARS: ( 2019)

### 1.2.11 PQDT: ProQuest Dissertations and Theses

(course*.ti,aby. OR syllabyus*.ti,aby. OR student*.ti,aby. OR teach.ti,aby. OR lesson*.ti,aby. OR feedback.ti,aby. OR classroom*.ti,aby. OR tutorial*.ti,aby. OR tutor*.ti,aby. OR seminar*.ti,aby. OR presentation*.ti,aby. OR practical*.ti,aby. OR online study*.ti,aby. OR fieldwork*.ti,aby. OR "face to face" .ti,aby. OR "online learning*" .ti,aby. OR "e learning*" .ti,aby. OR demonstration*.ti,aby. OR "computer based*" .ti,aby. OR "case studies" .ti,aby. OR "case study" .ti,aby. OR Clinical Competence/ OR training*.ti,aby. OR "clinical competenc*" .ti,aby. OR "clinical skill*" .ti,aby. OR Education/ OR "workshop*" .ti,aby. OR "training program*" .ti,aby. OR "educational activit*" .ti,aby. OR "competency based education" .ti,aby. OR Competency-Based Education/) AND (Cardiotocography/ OR "fetal monitor*" .ti,aby. OR "foetal monitor*" .ti,aby. OR cog.ti,aby. OR cardiotoco*.ti,aby. OR "foetal heartbeat*" .ti,aby. OR "fetal heartbeat*" .ti,aby. OR "foetal distress" .ti,aby. OR "fetal distress" .ti,aby. OR "foetal distress" .ti,aby. OR "fetal distress" .ti,aby. OR "late deceleration*" .ti,aby. OR "early deceleration*" .ti,aby. OR "foetal heart acceleration*" .ti,aby. OR "fetal heartrate" .ti,aby. OR "foetal heart rate" .ti,aby. OR "continuous electronic monitoring in labour" .ti,aby. OR "continuous electronic monitoring intrapartum" .ti,aby. OR "continuous electronic monitoring in delivery" .ti,aby. OR "foetal heart variability*" .ti,aby.)

## 2 Grey Literature searches

### 2.1 Search overview

Searches were originally run in December 2017 and updated to end Oct 2019. Note: These resources cannot be limited by year/date of publication, so total numbers for each search are listed, and results were de-duplicated against the original set of hits to obtain/identify new material.

| Table A2. Summary of grey literature searches | |
|---|---|
| Databases | No. of hits since December 2017 |
| Clinicaltrials.gov | 84 |
| opengrey | 94 |
| Grey Literature Report | 0 |
| NHS Evidence | 6078 |
| Total | 6467 |
| Deduplicated | 4170 – this is likely to be an over-estimate since the data/references exported from each resource is inconsistent. |

### 2.2 Search strategies

#### 2.2.1 OpenGrey (http://www.opengrey.eu/)

Cardiotocography OR fetal monitor* OR foetal monitor* OR CTG OR cardiotoco* OR foetal heartbeat* OR fetal heartbeat OR fetal heart rate OR continuous electronic monitoring in labour

#### 2.2.2 Grey Literature Report (https://www.nyam.org/library/collections-and-resources/grey-literature-report/)

Cardiotocography OR fetal monitor* OR foetal monitor* OR CTG OR cardiotoco* OR foetal heartbeat* OR fetal heartbeat OR fetal heart rate OR continuous electronic monitoring in labour

(Stopped adding new material in 2016, so no change for update)

#### 2.2.3 NICE Evidence Search (https://www.evidence.nhs.uk/)

Cardiotocography OR fetal monitor* OR foetal monitor* OR CTG OR cardiotoco* OR foetal heartbeat* OR fetal heartbeat OR fetal heart rate OR continuous electronic monitoring in labour

0 when searched as a string

| Table A3. NICE Evidence search results | |
|---|---|
| Cardiotocography | 290 |
| fetal monitor*<br>fetal monitoring | 0<br>2700 |
| foetal monitor*<br>“foetal monitoring”<br>fetal monitoring | 3028<br>28<br>2689 |
| CTG | 290 |
| Cardiotoco* | 63 |
| foetal heartbeat* | 115 |
| fetal heartbeat | 108 |
| fetal heart rate | 1778 |
| continuous electronic monitoring in labour | 734 |
| Total | 6078 |

# 3 Ongoing clinical trials register searches

### 3.1.1 Clinical trials (https://clinicaltrials.gov/)

Cardiotocography OR fetal monitor* OR foetal monitor* OR CTG OR cardiotoco* OR foetal heartbeat* OR fetal heartbeat OR fetal heart rate OR continuous electronic monitoring in labour

### 3.1.2 ICTRP (http://apps.who.int/trialsearch/)

Cardiotocography OR fetal monitor* OR foetal monitor* OR CTG OR cardiotoco* OR foetal heartbeat* OR fetal heartbeat OR fetal heart rate OR continuous electronic monitoring in labour
