# Supplementary material for: Training in the use of intrapartum electronic fetal monitoring with cardiotocography: systematic review and meta‐analysis
Source: BJOG. 2021 Jan 22;128(9):1408–19. doi: 10.1111/1471-0528.16619 (PMC8359372; doi:10.1111/1471-0528.16619)
Supplement: Supplementary file 4 — Appendix S2. Changes from study protocol. [file BJO-128-1408-s012.pdf]

Appendix S2. Changes from study protocol

We made two major changes between the study protocol and the review as conducted:

(1) The first related to the tool used to assess risk of bias for individual studies. We found that the MMAT tool was more relevant than the pre-specified Joanna Briggs critical appraisal tools to the types of the studies identified in the review. This was because MMAT was better able to incorporate the wide range of study designs included in the review.

(2) The second related to broader inclusion criteria for some studies. The protocol stated that: ‘studies describing or exploring the effect of training healthcare professionals to interpret and act on cardiotocography traces within a more complex or wider intervention (for example, studies that utilise two or more interventions aimed at changing health professionals' learning or behaviour), provided a discrete analysis of the CTG component is provided’ would be included. We found several studies that reported CTG as part of a much wider intervention. In these cases, a discrete analysis of the CTG component was not generally reported, but for completion of the overall range of studies we have summarised these in Appendix S3 (Section 2.6, Table C8).

References

MMAT: Hong QN, Pluye P, Fabreques S, et al. Mixed methods appraisal tool (MMAT) Version 2018. Registration of Copyright (#1148552), Canadian Intellectual Property Office, Industry Canada. Retrieved from http://mixedmethodsappraisaltoolpublic.pbworks.com/w/file/fetch/127916259/MMAT_2018_criteria-manual_2018-08-01_ENG.pdf. Date last accessed 21-5-2020.

Joanna Briggs critical appraisal tools: http://joannabriggs-webdev.org/research/critical-appraisal-tools.html
