# Supplementary material for: Training in the use of intrapartum electronic fetal monitoring with cardiotocography: systematic review and meta‐analysis
Source: BJOG. 2021 Jan 22;128(9):1408–19. doi: 10.1111/1471-0528.16619 (PMC8359372; doi:10.1111/1471-0528.16619)
Supplement: Supplementary file 5 — Appendix S3. Full data tables with individual study risk of bias assessments Contents [file BJO-128-1408-s003.pdf]

Appendix S3. Full data tables with individual study risk of bias assessments

Contents

1 The components and methods of delivery of CTG training 2

1.1 Table 1. Comparisons of components of training delivery 2

1.2 Table 2. Comparisons of one method of training delivery versus another method . 6

2 The impact of intrapartum CTG training on reactions, knowledge, behaviours, and maternal/fetal outcomes 9

2.1 Table 3. Reaction to CTG training (Kirkpatrick level 1) 9

2.2 Table 4. Learning assessed through test results (Kirkpatrick level 2) 14

2.3 Table 5. Learning assessed through inter-observer agreement of CTG interpretations (Kirkpatrick level 2) 29

2.4 Table 6. Learning assessed through performance in simulated scenarios (Kirkpatrick level 2)... 31

2.5 Table 7. Behaviours following CTG training (Kirkpatrick level 3).......................... 34

2.6 Table 8. Maternal/fetal outcomes following CTG training (Kirkpatrick level 4) 41

1 The components and methods of delivery of CTG training

1.1 Table C1. Comparisons of components of training delivery

Reference	Study design	Training 1	Training 2 (Comparator)	Study participants	Outcome measure	Results	Overall quality assessment
Quantitative randomised controlled trials							
Evans 1998	RCT	Instruction on selection of appropriate nursing interventions for the labouring woman experiencing FHM, with the inclusion of an analogy in a written self-instructional lesson (n=87 randomised overall) (41 analysed in the intervention group)	Instruction without an analogy but with same instruction on selection of appropriate interventions. This group were also provided background information on FHM in order to equalize reading time in both groups (39 analysed)	Nursing students	20 item criterion-referenced test - with two 10 item subtests, one for knowledge of concepts and one for application of rules	Outcome measure: mean test results<br><br>Knowledge of concepts:<br>After intervention (Test 1): 8.10 (SD 1.30)<br>Retention[1] (Test 2): 6.29 (SD 1.95)<br><br>Non-analogy control (Test 1): 8.62 (1.14)<br>Retention (Test 2): 7.08 (1.31)<br><br>Application of rules: After intervention (Test 1): 8.12 (SD 1.14)<br>Retention (Test 2): 7.15 (SD 1.57)<br><br>Non-analogy control (Test 1): 8.56 (SD 0.99)<br>Retention (Test 2):<br>7.85 (SD 1.39)<br><br>(statistical comparisons between means were not reported)	Low risk of bias

[1] Timing of this retention test is unclear as this paper was only available as a microfiche

Kinnick 1990	RCT	Teaching electronic fetal monitoring using all teaching variables in the Tennyson-Cochhiarella model (including labels and definitions, best examples, expository examples, and interrogatory examples) (n=68 were included overall) (67 students were included in the analysis of Test 1 and 61 for test 2) (numbers by treatment group were not reported)	Control 1: Modification of the Tennyson-Cochhiarella model; only some of the teaching variables prescribed in the model were used that were most similar to those used in traditional teaching (including labels, definitions, and presentation of best examples)<br><br>Control 2: Tennyson-Cochhiarella model; same as control group 1 plus an 'expository presentation'	Maternity nursing students	Evaluation of 18 EFM patterns	Outcome measure: mean test results<br><br>After intervention (Test 1):<br>Treatment group: 66.55 (SD 12.8)<br>6 to 7 weeks after clinical experience (Test 2): 74.1 (SD 10.4)<br><br>Comparison group 1: Test 1: 52.27 (SD 12.8)<br>Test 2: 66.4 (SD 13.2)<br><br>Comparison group 2: Test 1: 57.21 (SD 15.5)<br>Test 2: 65.2 (SD 14.3)<br><br>For Test 1, the intervention group had a significantly higher mean than the control groups (p=0.002). The difference was not significant for Test 2	High risk of bias
Wilson and Mires 2000	RCT (*the authors reported this*	Computer-assisted learning (CAL)	Computer-assisted learning (CAL) session	Medical students and midwifery	How to interpret a cardiotocograph – test score out of 24	Outcome measure: median test score<br><br>Computer-assisted learning group:	High risk of bias

	study as a pre-post study, but as half of the students were randomly allocated to a second intervention, we considered this study to be an RCT)	session (n=not clear; 38 medical students and 13 midwifery students included in the analysis)	plus a tutorial (n=not clear; 34 medical students and 11 midwifery students included in the analysis)	students (analysed separately)	(12 CTGs were interpreted)	*Medical students:*<br>Baseline test (Test 1): 9<br>After intervention (Test 2): 17 (p<0.001 within group comparison)<br><br>*Midwifery students:*<br>Baseline test (Test 1): 12<br>After intervention (Test 2): 14 (p=ns within group comparison)<br><br>Computer-assisted learning group plus tutorial:<br>*Medical students:*<br>Baseline test (Test 1): 8.5<br>After intervention (Test 2): 18 (p<0.001 within group comparison)<br><br>*Midwifery students:*<br>Baseline test (Test 1): 9<br>After intervention (Test 2): 16 (p=0.01 within group comparison)<br><br>Between group comparisons were not presented	
Wilson and Mires 2001	RCT (*the authors reported this study as a pre-post study, but as half of the students were randomly allocated to a second*	Computer-assisted learning (CAL) session –in four 2-hour sessions (n=not clear; 178 participants overall were included in the study) (45	Computer-assisted learning (CAL) session plus a tutorial (n=not clear) (50 in this group had both pre-and post-test data and were included in the analysis)	Medical students and midwifery students	CTG interpretation – students were provided with 12 photocopied CTGs to interpret with multiple choice answers. Marks were out of 24	Outcome measure: knowledge gain (%) (the authors stated that the data represent median values)<br><br>Computer-assisted learning group: 32%*<br><br>Computer-assisted learning group plus tutorial: 39%* (t=2.02, p=0.05)<br>*estimated from bar graphs	High risk of bias

	intervention, we considered this study to be an RCT)	in this group had both pre- and post-test data and were included in the analysis)					

1.2 Table C2. Comparisons of one method of training delivery versus another method

Reference	Study design	Training 1	Training 2 (Comparator)	Study participants	Outcome measure	Results	Overall quality assessment
Quantitative randomised controlled trials							
Murray and Higgins 1996	RCT	Lecture on FHR with transparencies (235 minutes) (n=20) (n=19 analysed)	Equivalent computer assisted instructional program (n=22) (n=20 analysed)	Nursing students given their first maternity rotation	Interpretation of 10 traces at pre-test (with 85 possible points) and 10 traces at post-test (with 72 possible points)	Outcome measure: mean % correct<br><br>Lecture group baseline (Test 1): 45.0%<br>After intervention (Test 2): 62.7%<br><br>Computer groups baseline (Test 1): 43.1%<br>After intervention (Test 2): 63.7% (p=ns)	High risk of bias
Wilson and Mires 1998	RCT	Teaching basics of cardiotocograph interpretation through a 45-minute tutorial. This tutorial covered the need for CTG, included slides of CTGs with a tutor pointing out the important features, and a review of real CTGs (n=5)	Teaching basics of cardiotocograph interpretation through a 45-minute session at the computer (n=4)	Midwifery students	Written test to assess CTG interpretation (full details not reported)	Outcome measure: correct answers (%)<br><br>Tutorial group baseline (Test 1): Baseline: 55%*<br>2 weeks after intervention (Test 2): 55%*<br><br>Computer training baseline (Test 1): 25%*<br>2 weeks after intervention (Test 2): 50%* (tutorial vs. computer p=0.65)	High risk of bias

						*an overall mean for each group of students was calculated based on data provided in figures (the authors presented the percentage of correct answers for each participant separately)	
Quantitative non-randomised							
Keegan et al. 2016	Quantitative nonrandomised	Students were given a traditional reading assignment as a pre-class learning activity (n=84) (n=30 included in analysis)	An active learning simulation of EFM on a mobile device, given as a pre-class assignment (n=32) (n=30 included in analysis)	Nursing students	EFM knowledge assessed by a 10-item quiz	Outcome measure: mean score (%)<br><br>After reading intervention: 70% (SD 28)<br><br>After mobile device intervention: 85% (SD 20.6) (p=0.01)	High risk of bias
Lee 2019	Quantitative nonrandomised	High-fidelity simulation training (n=36). Scenario-based simulations following a series of meetings. Five sessions of high-fidelity simulation were completed in 1 day with a video recording system, a pelvic model, and programmable fetal	Traditional didactic teacher-centred lecture (n=21). Six sessions of traditional teacher-centered lecture training were completed in the classroom. The lectures included (a) pathophysiology of maternal and fetal oxygen delivery, (b) uterine contraction	Nurses who worked in the labour and birth units for more than 3 months. All participants female.	Two standardised written tests (FHR knowledge and interpretation skill) and a self-reported questionnaire for perceived clinical management competence.	Outcome measure: mean score (%)<br><br><u>Knowledge</u><br>After intervention:<br>Simulation: 91.5+/-5 (range 80-100); p<0.001 for change from baseline (ch)<br>Didactic lecture: 89.5+/-5.2 (range 76-92); p=0.001 (ch)	Unclear risk of bias

		monitors. Delivered by five clinical experts, including multidisciplinary expertise and an outside consultant. The general debrief included group reflection of clinical implementation by using the videotape of the simulation and a facilitated discussion of par-ticipants' performance with trained faculty. Afterward, a 1-hour high-fidelity simulation was conducted every month for 3 months.	and placental function, (c) definition, evaluation, and record of FHR monitoring, (d) management of abnormal FHR, (e) effects of abnormal FHR, (f) and discussion (Gall et al., 1985). Afterward, a 1-hour lecture was conducted every month for 3 months. The contents of lectures were de-signed according to the recommendations of the American College of Obstetricians and Gynecologists (2009).			Skills<br>After intervention:<br>Simulation: 90.8+/-5.8 (range 76-100); p<0.001 for change from baseline (ch)<br>Didactic lecture: 80.8+/- 8.4 (range 70-94); p=0.097 (ch)<br><br>(between group comparison not reported)<br><br>Perceived competence in clinical management: p<0.05 for all measures immediately after training and 6 months later.	
O'Boyle 1995	Quantitative nonrandomised	Structured workshop (6 hours) (n=30) (all included in the analysis)	Learning by videotape (n=30) (all included in the analysis)	Obstetric nurses	There was a maximum of 85 points for the pre-test while the post-test had a maximum of 72 points (to assess the ability to interpret EFM monitor strips)	Outcome measure: mean gain of achievement percentile scores (comparing pre-test to post-test)<br><br>After workshop: 10.6 (SD 8.0)<br>After video: 7.6 (SD 5.4) (p=0.10)	Low risk of bias

2 The impact of intrapartum CTG training on reactions, knowledge, behaviours, and maternal/fetal outcomes

2.1 Table C3. Reaction to CTG training (Kirkpatrick level 1)

Reference	Study design	Training	Comparator	Study participants	Outcome measure	Results	Overall quality assessment
Quantitative randomised							
Wilson 2001	Quantitative randomised (the authors reported this study as a pre-post study, but as half of the students were randomly allocated to a second intervention, we considered this study to be an RCT)	Computer-assisted learning (CAL) session –in four 2-hour sessions (n=not clear; 178 over all were included in the study) (45 in this group had both pre-and post-test data and were included in the analysis)	Computer-assisted learning (CAL) session plus a tutorial (n=not clear) (50 in this group had both pre-and post-test data and were included in the analysis)	Medical students and midwifery students	"Attitude and enjoyment" of students with "computer assisted instruction"<br>Staff "enjoyment of teaching"	Outcome measure:<br>Students:<br>"Did you enjoy the programme" (Figure 5)<br>Only graph image available (approx. 45% students 5/5 "yes very much"; 47% 4/5 n=105)<br><br>Staff:<br>Not reported	High risk of bias
Beckley 2000	Quantitative randomised	Computer-assisted teaching programme<br><br>Overall sample size n=117 (115 completed Test 2)	No programme (participants) received programme later)	Midwives and obstetricians	Opinion of teaching programme (rate 1-5 agree; disagree) (e.g. "I enjoyed the package; the diagrams helped to explain the text)	Outcome measure:<br>Page 1141 Table 2<br>Scores on an opinion questionnaire were similar between the groups relating to the overall impression of the teaching package, the usefulness of the package, the ease of	Unclear risk of bias

						use, and presentation of the material.	
Quantitative descriptive							
Kroushev 2009	Quantitative descriptive	All day workshop – physiology, CTG interpretation and management	N/A	Obstetricians, midwives, medical students and GPs	Course feedback ("helpfulness and usefulness of course")	Page 269 Table 1 *"Almost all participants reported that they either 'strongly agreed' or 'agreed' that the program was relevant to their practice and that it improved their knowledge and understanding of IFS. Of all the questions asked, participants were least certain that the workshop had increased their confidence in interpreting cardiotocographs* (49% = strongly agree)"	High risk of bias
Ren 2017	Quantitative descriptive	Medical simulation models (including fetal heart rate monitoring) (n=80)	N/A	Medical students	Satisfaction with simulation model course	Page 998, Table 1<br><br>100% (N=80) wish to continue participating in this program	High risk of bias
Burke 2013	Quantitative descriptive	2 hour didactic and simulation session (n=372)	N/A	Obstetricians, midwives, residents, anaesthesiologists and nursing staff	Participant ("providers and nurse") reaction at end of session	"97% rated the overall programme as excellent"	Low risk of bias
Catanzarite 1987	Quantitative descriptive	Computer aided instructional	N/A	Medical students	"Reactions" of nurse educators and obstetrics faculty	Faculty/Educators:	Low risk of bias

		programme (participant numbers not reported)			Post session questionnaire to medical students	"reactions uniformly positive"<br>Students:<br>"first six groups...ratings were favourable...virtually all found FMTUTOR to be a valuable teaching aid..."	
Haire 1978	Quantitative descriptive	"trained nurse... in interpretation of tracing" delivering all day "sessions" – no more detail provided (n=150 responses provided – unclear how many actually participated)	N/A	Nurses, physicians	Opinions and suggestions on the educational programme (questionnaire – 4 questions – response = yes/no/occasionally)	Page 30, Table 1<br>Was the information clearly presented? 144/150 yes;<br>Was the information applicable to your situation? 126/150 yes;<br>Were the suggestions for treatment practical? 133/150 yes;<br>Were the sessions a learning experience for you? 140/150 yes	Unclear risk of bias
Votaw 1979	Quantitative descriptive	Computer aided "development and assessment" of competence in fetal monitoring (Participant numbers not reported)	N/A	Midwives, medical students, nursing students, residents, physicians	Feedback from participants *"diagnostic and pilot testers regarding their subjective reactions to the course segments"*	No Figures provided<br>*"...in response to positive assertions concerning clarity of objectives, content accuracy, currency and presentation, quality of questions and feedback, ease of access to the lessons, etc., the mean response for nurses fell in the agree, strongly agree range on a four-point*	Unclear risk of bias

						scale. Positive assertions concerning the unique nature of the learning experiences and a preference for the computer-based approach received an equally favourable response."	
Knight 2019 (Abstract)	Quantitative descriptive	A structured CTG training approach, using both familiar FIGO and new physiological guidelines. "introductory half day multidisciplinary teaching session on CTG physiology". Weekly 60 minutes CTG sessions led by consultant/ST6	None	Obstetric consultants, junior doctors, "midwifery practice leaders"; midwives	Staff survey 6 weeks after training, covering understanding and feedback.	Presession confidence was greater about FIGO (9% extremely, 49% confident, 34.5% somewhat, 7.5% not) compared with physiological interpretation (5.7%, 34.2%, 40.3%, 19.6%). Postsession confidence increased in physiological (36% strongly, 62% agree, 2% disagree) with almost all finding the session useful (39.5% extremely useful, 54.7% very useful, 5.7% somewhat useful, 0% not useful).	High risk of bias
Quantitative nonrandomised							
Keegan 2016	Quantitative nonrandomised	An active learning simulation of EFM on a mobile device, given as a	Students were given a traditional reading assignment as a	Nursing students	Student feedback on using the mobile device simulation of EFM (questionnaire – Likert scale)	Page 58 Table 2 24/40 (60%) agree – "it was more helpful to learn EFM concepts by	High risk of bias

		pre-class assignment (n=32) (n=30 included in analysis)<br><br>(n=40* in responses to survey)	pre-class learning activity (n=84) (n=30 included in analysis)			using the EFM app than by reading”<br>35/40 (88%) agree – “The EFM mobile simulation was a useful approach to learning”<br><br>*participant numbers responding to survey on EFM app exceeded number randomised to EFM arm	

2.2 Table C4. Learning assessed through test results (Kirkpatrick level 2)

Reference	Study design	Training	Comparator	Study participants	Test type	Results	Individual study risk of bias (MMAT)
Quantitative randomised controlled trials							
Beckley et al. 2000	RCT	Computer-assisted teaching programme<br><br>Overall sample size n=117 (115 completed Test 2)	No programme (participants) received programme later)	Midwives and obstetricians	Test consisting of 80 multiple choice questions (to test knowledge of acid-base balance and cardiotocography)	Outcome measure: mean test scores (%)<br><br>Intervention group baseline (Test 1): 50.5% After intervention (Test 2): 70.2%<br><br>Control group baseline (Test 1): 50.3%<br>No intervention (Test 2): 54.8% (p<0.001)	Unclear risk of bias<br><br>*Note – subgroup analysis by type of healthcare worker was also presented*
Carbonne and Sabri-Kaci 2016	RCT	E-learning training programme (n=57) (35 completed test 2)	No training (n=56) (28 completed test 2)	Midwives (or student midwives) and obstetricians	Web-based certification test with points up to 50 (to test knowledge in cardiotocography analysis)	Outcome measure: mean test scores (out of 50)<br><br>Intervention group baseline (Test 1): 32.5 (SD 4.6)<br>After intervention (Test 2): 37.1 (SD 5.5)<br><br>Control group baseline (Test 1): 32.4 (SD 5.2), (p=0.989)<br>No intervention (Test 2): 32.6 (SD 5.7), (p=0.0026)	High risk of bias<br><br>*Note – subgroup analysis by type of healthcare professional was also presented*

Devane et al. 2006	RCT	1.5 hr fetal monitoring lecture (n=27) (all included in the analysis)	Alternative education programme consisting of a non-fetal-monitoring-related video presentation (n=28) (all included in the analysis)	Midwives	Multiple-choice test with 16 questions on fetal monitoring knowledge and 6 multiple-choice items for each of three tracings (to test knowledge and skills)	Outcome measure: median % correct<br><br>Fetal monitoring knowledge test:<br>Intervention group baseline (Test 1): 63% (IQR 69, 50)<br>After intervention (Test 2): 88% (IQR 94, 81)<br><br>Control group baseline (Test 1):<br>63% (IQR 69, 44)<br>No intervention (Test 2): 56% (IQR 69, 50)<br>(mean difference: 32%, U=78.5, 95.1% CI: -31.3, -18.8, p<0.001)<br><br>CTG interpretation skills:<br>Intervention group baseline:<br>61% (IQR 67, 50)<br>After intervention (Test 2): 67% (IQR 78, 56)<br><br>Control group baseline: 56% (IQR 67, 44)<br>No intervention (Test 2): 56% (IQR 67, 50)<br>(mean difference:	High risk of bias

						11%, U = 186, 95.2% CI: -16.7, -5.6, p<0.001)	
Rizk and Hafez 2013	RCT	Education using both didactic methodology and a computer-based childbirth simulator (n=100) (all participants included in the analyses)	Nurses in the control group received the routine in-service training which involved orientation about how to operate the machine and monitor information (n=100) (all participants included in the analyses)	Maternity nurses	Test consisting of 25 items covering seven categories (to test knowledge of EFM)<br><br>*Given the number of categories reported, we have selected one category to include in the meta-analysis: 'general knowledge about EFHM' (one month after the intervention) as it appears to be a main outcome*	Outcome measure: correctly answered questions (%)<br><br>Intervention group baseline (Test 1): 17/00 (17%)<br>1 month after intervention (Test 2): 52/100 (52%)<br>3 months after intervention (Test 3): 62/100 (62%)<br><br>Control group baseline (Test 1): 17/100 (17%) (no significant difference between intervention and control)<br>1 month after no intervention (Test 2): 16/100 (16%) (X^2=0.632, p<0.0001)<br>3 months after no intervention (Test 3): 15/100 (15%) (X^2=0.632, p<0.0001)	Unclear risk of bias
Trépanier et al. 1996	RCT	One day fetal monitoring education programme – with a half day review session 6 months	No programme (participants) received programme later) (n=62 nurse	Nurses (with intrapartum care responsibilities)	Two instruments were used: a knowledge test with 45 multiple-choice questions and a 25-item performance test (to evaluate clinical skills	Outcome measure: mean % correct<br><br>Knowledge test:	Unclear risk of bias

		afterwards (n=47) (all included in first two tests, 40 in third test)	participants) (all included in first two tests, 56 in third test)		through four simulated case scenarios)<br><br>*We have selected one category to include in the meta-analysis: 'knowledge test' (immediately after the intervention) as is most similar to other studies included in the meta-analysis*	Intervention group baseline (Test 1): 62.6% (SD 12.97)<br>After intervention (Test 2): 75.3% (SD 8.14)<br>6 months after intervention (Test 3): 74.3% (SD 12.38)<br><br>Control group baseline (Test 1): 60.5% (SD 13.93)<br>No intervention (Test 2): 59.4% (SD 10.96)<br>6 months with no intervention (Test 3): 65.1% (SD 12.79)<br>(Interaction of time by group: *F* [3, 216] = 21.40, p<0.001, Greenhouse-Geisser corrected probability)<br><br>Skills test:<br>Baseline: no data<br>After intervention (Test 2): 91.7% (SD 6.49)<br>6 months after intervention (Test 3): 82.2% (SD 8.83) (this 9.5% decrease within the intervention group was significant; p<0.001)	

						Baseline: no data<br>Control with no intervention (Test 2): 74.0% (SD 12.67)<br>6 months with no intervention (Test 3): 71.8% (SD 12.19)<br><br>No statistical results were reported for the skills test	
Quantitative non-randomised studies							
Cook 2015 (Abstract)	Quantitative nonrandomised	Training involving 240 hours of didactic and practical instruction (n=2)	NA (pre-post)	Nurses (general) from community hospitals who have a low volume of births	The authors only stated that the Association of Women's Health, Obstetric and Neonatal Nurses' Perinatal Orientation and Education Program (PEOP) testing was used (to assess knowledge)	Outcome measure: mean test scores (%)<br><br>The authors stated that "scores on the POEP post-tests increased by 32% and 44%, respectively, with an overall average score of 98.5% at the conclusion of the training."	Unclear risk of bias
Cooke et al. 2010	Quantitative nonrandomised	On-line training plus two-day face-to-face education (i.e. train-the-trainer education programme) (n=240 trainers trained 954 clinicians) (sample sizes included in the analysis were not reported)	NA (pre-post)	Clinicians who were trained by those who received the intervention	Assessment and grading of two EFM patterns (one normal and one pathological)<br><br>*For each of these patterns, the % of correct responses were reported for 1) baseline fetal heart rate, 2) variability, 3) accelerations, 4) decelerations and 5)*	Outcome measure: correct assessments (%)<br><br>Normal EFM pattern:<br>Baseline test (Test 1): 64%*<br>After intervention (Test 2): 67%*	Unclear risk of bias

					overall diagnosis. We *have selected one category: 'overall diagnosis' to report here as it appears to be the most informative outcome*	The authors stated, "For the normal EFM pattern…the improvements were in their identification and definition of variability (p=0.02), accelerations (p=0.033) and decelerations (p=0.003)."<br><br>Pathological EFM pattern:<br>Baseline test (Test 1): 59%<br>After intervention (Test 2): 77%* (p=0.005)<br><br>The authors stated "For the pathological EFM pattern, there was a significant improvement in the clinicians' ability to diagnose the pathological nature of the pattern (P = 0.005). Specifically, the understanding of the components of the pathological pattern namely baseline heart rate (P = 0.004), variability (P = 0.023), accelerations (P =	

						0.001) and decelerations (P = 0.0000)."<br><br>*data were extracted from bar graphs using Webplotdigitizer	
Daglar 2019	Quantitative nonrandomised	Practical individual and group training on Trace Interpretation, Fetal Circulation and Asphyxia, the Non-stress Test (NST), Intrapartum Monitoring, Assessment Criteria, and Responsibilities of Midwives in Non-reactivity.<br><br>(n=103 (42 in 2015 and a different 61 in 2016)	None (pre-post)	Final year midwifery students	Electronic Fetal Monitoring Course Pre-test including 14 questions on the theoretical knowledge of and skills to interpret the EFM and 10 traces, the “Electronic Fetal Monitoring Course Post-test” including similar questions, and the “Trace Interpretation Competence Criteria” including 10 criteria.	Mean test scores: Before training (n=103) 55.29+/=11.17; After training (n=102) 76.15+/= 6.75; p=0.030	Low Risk of bias
Froc 2018	Quantitative nonrandomised	In situ training program on CTG interpretation during labour in 15 hospital based maternity units. Theoretical teaching by a pair of expert midwives-obstetricians, using a	None	Midwives, obstetricians, "DES interns"	Mean test scores before (T0), immediately after (T1) and median 40 months after training (T2). Assessed using a questionnaire based on 10 questions based on the Colle's RCF Classification with ten single-choice questions, both theoretical based on fetal heart rate	Mean test scores: T0 4.79 (95% CI 4.54, 5.02); T1 6.71 (95% CI 6.49, 6.93), p<0.05; T2 5.32 (95% CI 4.94, 5.70), p<0.001.	High risk of bias

		participant knowledge assessment questionnaire with theoretical training (using standard classification CNGOF (French)) and practical training using 30 traces. Plus theoretical reminder of the basic rules of RCF surveillance (importance of calls for advice, cross-monitoring, medical aspect, communication within schools, team).<br><br>n=234 (T0); 228 (T1)			classification by the CNGOF, and evidence-based clinical record practices to be analysed.		
Jomeen 2019	Quantitative nonrandomised	CTG training in the community. Multiprofessional training in cardiotocograph (CTG) interpretation delivered by a social enterprise organisation (Baby Lifeline Training Ltd). Face-to-face lecture-based day aimed at providing evidence-based training on CTG interpretation.<br>n=255	None	NHS community midwives,specialist midwives, midwifery managers, labour ward coordinators,student midwives, obstetricians, paramedics, and student paramedics	Practitioner knowledge, confidence, and empowerment immediatelypretraining and posttraining and at 12 weeks following training.<br><br>Empowerment:<br>A pretraining and posttraining measure, with 20items, A four-question delegate satisfaction form.<br>Knowledge: A 10-item true/false questionnaire<br>Confidence: A 12-item self-assessed pre and post	Pre-test; post-test; 3 month follow-up.<br>Knowledge re: CTG interpretation: increased from 2.57 (0.59); 3.31(0.53); 3.00 (0.59); T1 v T2 <0.001; T1 v T3 <0.01.<br>Confidence re: CTG interpretation: 2.53 (0.70);3.25 (0.55); 2.89 (0.63); T1 v T2 <0.001; T1 v T3 0.001.<br>Confidence re: clinical situations CTG interp: 2.56 (0.80); 3.18	Unclear risk of bias

					questionnaire which assessed participant confidence in the main components of the course.	(0.56); 2.87 (0.65); T1 v T2 <0.001; T1 v T3 0.002. Confidence in leading change: 2.03 (0.93); 2.88 (0.73); 2.19 (0.99); T1 v T2 <0.001; T1 v T3 0.05. CTG Knowledge test score: 5.51(1.64); 8.07 (1.78); 6.85 T1 v T2 <0.001; T1 v T3 <0.001. Also improved confidence in understanding 'the types of intrapartum hypoxia and the resultant features observed on a CTG trace' (p=0.04); 'control of fetal heart (p=0.004)'; 'fetal pathophysiology p=0.005'; however confidence in understanding of 'wider clinical picture' and 'national guidelines' not significant.	
Mahley et al. 1999	Quantitative nonrandomised	Didactic presentation (2 hours) followed by the use of EFM process cards on the clinical units (n=116)	Pre-post (Before use of cards was introduced) (n=59) (Two years before	Nursing students	Seven multiple-choice questions (to test knowledge and interpretation of one EFM tracing)	Outcome measure: mean correct response across seven questions (%)	High risk of bias

			implementation of intervention)		*The % of correct responses for each question have been reported in the paper. We have chosen one question to focus on in our analyses: 'Appropriate nursing interventions when the nurse sees late decelerations on the fetal monitor are...?' as this question appears to best assess overall knowledge*	Baseline test (Test 1): 76%<br>After intervention (Test 2): 92% (p=0.003)	
Millde-Luthander et al. 2012	Quantitative nonrandomised	Computer assisted learning programme for interpreting CTG patterns (n=179)	NA (Pre-post)	Midwives and physicians	Proportion of individuals who correctly classified a randomly chosen paper-copy of a 40 min long CTG - from a sample of 40 (to assess ability to interpret cardiotocography)	Outcome measure: correct classifications (%)<br><br>Baseline test (Test 1): 115/179 (64%)<br>Approx. 1-32 days after intervention (Test 2): 89/135 (66%) (p=0.76)	High risk of bias<br><br>*Data also reported by type of healthcare professional – and by type of CTG*
Miller and Miller 2013	Quantitative nonrandomised	Train the trainer course on EFM (1.5 days in length) (n=not reported; the authors stated "close" to 400 participants completed the training sessions")	NA (Pre-post)	Nurses, midwives, and physicians (who would become trainers)	Multiple-choice questions (to test knowledge of EFM)	Outcome measure: mean correct response (%)<br><br>Baseline test (Test 1): 49%<br>After intervention (Test 2): 85%<br>6 months after intervention (Test 3): 80% (42% response rate)<br>18 months after intervention (Test 4):	High risk of bias

						84% (30% response rate)	
Stohl and Miller 2016	Quantitative nonrandomised	Teaching in FHR monitoring (n=8)	NA (pre-post)	Obstetrics and gynaecology physicians	Physician knowledge of the 2008 National Institute of Child Health and Human Development (NICHD) nomenclature using multiple-choice and short-answer tests	Outcome measure: mean test scores (%)<br><br>Baseline test (Test 1):<br>Multiple-choice: 73% (SD 18)<br>Short-answer: 47% (SD 13)<br><br>3 weeks after intervention (Test 2):<br>Multiple-choice: 97% (SD 5), p=0.003<br>Short-answer: 76% (SD 13), p=0.0001<br><br>6 months after intervention (Test 3):<br>Multiple-choice: 97% (SD 4)<br>Short-answer: 76% (SD 7) (p value not reported)<br><br>12 months after intervention (Test 4):<br>Multiple-choice: 96% (SD 4)<br>Short-answer: 75% (SD 10) (p value not reported)	High risk of bias
Thellesen et al. 2017	Quantitative nonrandomised (the authors	The implementation of a national CTG education programme	NA (pre-post)	Gynaecologists, obstetricians, general	10-item CTG multiple choice test (to test knowledge,	Outcome measure: % of participants who	Low risk of bias

/Thellesen et al 2019	described the 2017 study as a cross-sectional study, but pre-post data were also reported)	(1-day course) (n=1641, but pre-test/post-test data based on n=790)<br><br>Patient/delivery outcomes (Thellesen 2019)<br><br>All intended vaginal deliveries in Denmark resulting in a liveborn singleton in ce-phalic presentation with a gestational age≥37weeks.		specialists, residents, midwives	interpretation skills and decision-making)	improved their test scores<br><br>Baseline test (Test 1): 790/1641 (48.1%)* had test scores from 0-9<br><br>Immediately after intervention (Test 2): The authors appear to report that of 790 course participants who scored 0-9 on the test before implementation, 662 (83.8%) improved their score in the post-test (immediately after the course) (no statistical comparisons were reported)<br><br>*data calculated from figures reported in the paper<br><br>Patient/delivery outcomes (Thellesen 2019)<br><br>Pre-implementation; post-impl; 3 month follow-up:	

						5 min Apgar score<7: ref; 0.97(0.84, 1.11); 0.99 (0.90, 1.10); Emergency caesarean: ref; 1.05 (1.01, 1.08); 0.98 (0.96, 1.01) Assisted vaginal delivery: ref; 0.91 (0.87-0.95); 0.86 (0.84,0.89); Umbilical cord pH <7.00 and neonatal therapeutic hypothermia ns at both time periods.	
Parsons 2013 (Abstract)	Quantitative non-randomised	One-to-one education with 3 live tracings. 10 questions were asked regarding their interpretation of the tracings and they were asked to provide an explanation of how they would intervene and communicate information about the tracings. Follow-up remediation and education for any incorrect responses given.<br>(N=25)	NA (pre-post)	Nurses	Individual performance for the interpretation and management of three CTG traces rated on a 0-4 Likert scale based on the number of correct responses during their one to one session.	The average score of the 25 nurses in January 2012 was 1.68/4 (pre-education). The average score in March 2012 of the same 25 nurses was 2.4/4 (post-education). The average score in September 2012 was 3.65/4 (follow-up).	High risk of bias
Di Lieto 2002	Quantitative non-randomised	Linked “telematically” to a central operating unit to obtain	NA (pre-post)	Unclear	Evaluation questionnaire. The questionnaire included one CTG trace and 5 multiple	The average score in the questionnaire was 9 at 10 months from	High risk of bias

		computerised analysis of CTG traces and specialist consulting. Staff in outpatient units received theoretical-practical training in the function and use of the telemedicine system (TOCOMAT), guidelines for CTG traces interpretation, and diagnostic-therapeutic protocols. (Number of participants not reported)			choice questions, with a final possible score of between 0 and 15.	the beginning of the study, and 12 at the end of the study.	
Pettker 2011	Quantitative non-randomised	EFM training and certification, as part of a broader patient safety programme (see Pettker 2009).	N/A (Pre-post)	Nurses, residents. *'All medical staff members and employees who are responsible for fetal monitoring interpretation were obligated to take this examination.'*	success rate at the EFM certification test	*'There was a 100% pass rate among physicians and midwives and a 98% pass rate on first attempt among nurses over the time of this study.'*	High risk of bias
Quantitative descriptive studies							
Rehling-Anthony et al. 2011 (Abstract)	Quantitative descriptive (Prevalence study)	The implementation of standardized education and competence validation in EFM in a multihospital system (n=105) in 2009	NA	Labour and delivery nurses	Exam to obtain EFM certification (no details provided)	Outcome measure: pass rate (%)<br><br>After intervention: 93/105 (89%) in 2009	High risk of bias

Guild 1994	Quantitative descriptive	Fetal monitoring computer tutorial<br><br>Skills experience and assessment<br><br>(Number of participants not stated)	NA	Nurses	six CTG strip assessments with a score of at least 90%<br>25-item multiple choice test with a score of at least 80%	97% of obstetric nurse staff and leadership successfully attained recredentialing (passing the post-test) with an average score of 92%."	Low risk of bias

2.3 Table C5. Learning assessed through inter-observer agreement of CTG interpretations (Kirkpatrick level 2)

Reference	Study design	Training	Comparator	Study participants	Outcome measure	Results	Overall quality assessment
Quantitative non-randomised studies							
Ayres-de-Campos et al. 2004	Quantitative nonrandomised	'Teaching' FHR diagnostic criteria using a baseline definition proposed by the study authors, described as: "Three clinicians using the definition proposed by us, familiarised with its physiological basis, and where a previous training session to evaluate 10 difficult cases was promoted." (n=3)	Clinicians used the definition of the FIGO guidelines, which they routinely employed in their clinical practice (usual practice) (n=3)	Clinicians	FHR baseline estimation of 300 tracings; Statistical agreement between 3 observers in each group measured three ways: kappa; proportions of agreement; ICC coefficient)	Outcome measure: within group inter-observer agreement (using kappa[2])<br>Training group: 0.967 (0.941-0.994)<br>No training: 0.884 (0.858-0.910)<br>(no between group p values were reported)	High risk of bias
Davis et al. 2010 (Abstract)	Quantitative nonrandomised (obtained using a retrospective chart review)	Regular auditing by a team of staff nurses (including instructors). When auditors disagree with a case's EFM recognition or intervention, the case is forwarded to	NA (Pre-post)	Nurses	FHM interpretation (each month 40 charts, 20 labour inductions and 20 Pitocin augmentations were audited); Proportions of agreement between auditors and nurses	Outcome measure: proportions of agreement between auditors and nurses<br><br>The authors stated: "From the July 2008 baseline of 54% agreement, a steady	High risk of bias

[2] Kappa was chosen to report as it was most commonly reported in all of the studies

		one of two Advance Practice Nurses (APNs). The APNs perform a second review and collaborate with unit management for any identified learning needs (n=not clear)				improvement was noted through 2008 and 2009." Further data were not reported in this conference abstract	
Govindappagari et al. 2016	Quantitative nonrandomised	Mandatory 9-hour online training in management and interpretation of EFM (n=351 before and n=350 after)	NA (Pre-post)	Nurses	EFM interpretation 0f 701 charts at two time points (time of admission and before delivery); Proportions of agreement between obstetrical care providers (i.e. physician assistants, residents etc. in the obstetric department) and obstetric nurses<br>.	Outcome measure: proportions of agreement between providers and nurses<br><br>Baseline (June 1, 2009 to Dec 31, 2009): 84.3% agreement[3] at the time of admission, and 65.2% prior to delivery<br><br>After intervention (1 Dec 2012 to 30 July 2013): 92.3% agreement at the time of admission (p=0.001), and 74.0% prior to delivery (p=0.01)	Unclear risk of bias
Quantitative descriptive studies							
Blix and Øian 2005	Quantitative Descriptive	CTG mixed training programme (6-hour classroom and	None	Midwives and obstetricians	Classification of CTG - normal/intermediary/abnormal (549 traces); Statistical agreement	Outcome measure: k statistic and proportions of	High risk of bias

[3] Average agreement which included variability, accelerations and decelerations (data were also reported for each of these parameters separately).

	(Cross-sectional Survey)	theoretical lessons) (n=6)			between pairs of observers measured using k statistic	agreement between participants<br>Between the pairs of observers, k varied from 0.57 to 0.75; proportion of agreement for a normal labour admission test varied from 0.78 to 0.88, and for an intermediary/abnormal test varied from 0.56 to 0.69	

2.4 Table C6. Learning assessed through performance in simulated scenarios (Kirkpatrick level 2)

Reference	Study design	Training	Comparator	Study participants	Outcome measure	Results	Overall quality assessment
Randomised controlled trials							
Fransen et al. 2013	Cluster-RCT	Team training involving a 1-day course in a medical simulation centre (n=12 hospitals)	No training (n=12 hospitals)	Teams consisted of a gynaecologist, a midwife, a resident and two or three nurses. Each team was formed around a single gynaecologist.	Team performance as measured using the clinical teamwork scale (CTS) and acquisition of medical technical skills. This was measured by an in situ clinical simulation for two scenarios (delivery of baby with shoulder dystocia in the maternal all-fours position and conducting a perimortem	Outcome measure: median clinical teamwork score (overall score)<br><br>Intervention: 7.5, range 2.0–8.5<br><br>Control: 6.0, range 2.0–8.0 (p=0.01)	Low risk of bias

					caesarean section within 5 minutes for the scenario of amniotic fluid embolism)	Medical technical skills: The use of predefined obstetric procedure was performed in 19 of the 23 in situ simulation recordings (83%) in the trained units compared with 10 of the 22 recordings (46%) in the untrained units (p=0.009)	
Rizk and Hafez 2013	RCT	Education using both didactic methodology and a computer-based childbirth simulator (n=100) (all participants included in the analyses)	Nurses in the control group received the routine in-service training which involved orientation about how to operate the machine and monitor information (n=100) (all participants included in the analyses)	Maternity nurses	Checklist consisting of 15 items to assess nurses' performance in relation to the application, interpretation, and interventions of deviating FHR patterns (out of a total score of 30)	Outcome measure: proportion of nurses with a satisfactory performance score (≥15)<br><br>Intervention group baseline (Test 1): 14/100 (14%)<br>1 month after intervention (Test 2): 82/100 (82%)<br>3 months after intervention (Test 3): 83/100 (83%)<br><br>Control group baseline (Test 1): 18/100 (18%) (no significant difference	Unclear risk of bias

						between intervention and control)<br>1 month after no intervention (Test 2): 15/100 (15%) (X^2=89.86, p=0.0001)<br>3 months after no intervention (Test 3): 20/100 (20%) (X^2=79.45, p=0.0001)	
Cuerva 2018	RCT	Simulation-based training with briefing session in which teachers demonstrated a properly performed childbirth scenario that included CTG training as part of much wider maternity training.<br><br>However separate evaluation of CTG component so	Simulation training without demonstration of properly performed scenario)	Undergraduate medical students	Evaluation of performance assessed by teachers using a 5 point scale (separate CTG evaluation)	No significant differences between groups by simulation or debriefing session. (N=12 for each group). Simulation: Gp1 2.83+/- 0.718; Gp2 2.17+/-0.835. Debriefing: Gp1 2.83+/- 0.718; Gp 2 3.58+/- 0.90.	High risk of bias

		groups can be compared.					

2.5 Table C7. Behaviours following CTG training (Kirkpatrick level 3)

Reference	Study design	Training	Comparator	Study participants	Outcome measure and results	Overall quality assessment
Quantitative non-randomised studies						
Cook 2015 (Abstract)	Quantitative non-randomised	Training involving 240 hours of didactic and practical instruction (n=2)	NA (Pre-post)	Nurses (general) from community hospitals who have a low volume of births	The authors stated that "upon completion of the program, the nurses reported… feeling much better prepared to care for perinatal patients."	Unclear risk of bias
Davis et al. 2010 (Abstract)	Quantitative non-randomised: Pre-post (obtained using a retrospective chart review)	Regular auditing by a team of staff nurses (including instructors). When auditors disagree with a case's EFM recognition or intervention, the case is forwarded to one of two Advance Practice Nurses (APNs). The APNs perform a second review and collaborate with unit management for any identified learning needs (n=not clear)	NA (Pre-post)	Nurses	The authors stated that "another result [of the programme] has been a change in culture resulting in keener awareness and patient safety and collegial discussions of FHM tracings."	High risk of bias
Gnanasambambanthan 2018 (Abstract)	Quantitative non-randomised	Structured education programme for midwives and doctors including mandatory CTG	None	Midwives; doctors	Documentation of CTG interpretation improved from an average of 69% to 80%. 'Fresh-eyes' approach where EFM	High risk of bias

		masterclasses, weekly teaching with trained midwives and obstetricians<br><br>N of healthcare professionals participating not reported.<br>36 patient's charts audited "1% of total deliveries"			interpretation is checked by a second HC professional every 2 hrs improved from 11% to 60%. An action plan following local guidelines was implemented in 95% of cases where the CTG trace was assessed as suspicious or pathological, compared with 91% previously.	
Grace 2018 (Abstract)	Quantitative non-randomised	Interactive training in fetal heart rate monitoring, labor management and teamwork	None	Not reported	1 day workshop. Small interdisciplinary groups. Case studies for plans of care; emergency CS simulations, cervical exam and vaginal births, interpretation of electronic fetal monitoring.<br><br>Reported increase in "confidence in ability and understanding in most subject areas".<br>"improvements in safe functioning on lab/delivery unit, interpretation of EFM and intervention, use of communication tools and participation in emergency CS. Measured by survey before and after program.	High risk of bias

Jomeen 2019	Quantitative nonrandomised	CTG training in the community.<br>Multiprofessional training in cardiotocograph (CTG) interpretation delivered by a social enterprise organisation (Baby Lifeline Training Ltd).<br>Face-to-face lecture-based day aimed at providing evidence-based training on CTG interpretation.<br>n=255	None	NHS community midwives,specialist midwives, midwifery managers, labour ward coordinators,student midwives, obstetricians, paramedics, and student paramedics	Practitioner knowledge, confidence, and empowerment immediatelypretraining and posttraining and at 12 weeks following training.<br><br>Empowerment:<br>A pretraining and posttraining measure, with 20items, A four-question delegate satisfaction form. Knowledge: A 10-item true/false questionnaire Confidence: A 12-item self-assessed pre and post questionnaire which assessed participant confidence in the main components of the course.<br><br>Pre-test; post-test; 3 month follow-up. Knowledge re: CTG interpretation: increased from 2.57 (0.59); 3.31(0.53); 3.00 (0.59); T1 v T2 <0.001; T1 v T3 <0.01. Confidence re: CTG interpretation: 2.53 (0.70);3.25 (0.55); 2.89 (0.63); T1 v T2 <0.001; T1 v T3 0.001. Confidence re: clinical situations CTG interp: 2.56 (0.80); 3.18 (0.56); 2.87 (0.65); T1 v T2 <0.001; T1 v T3 0.002. Confidence in leading change: 2.03 (0.93); 2.88 (0.73); 2.19 (0.99); T1 v T2 <0.001; T1 v T3	Unclear risk of bias

					0.05. CTG Knowledge test score: 5.51(1.64); 8.07 (1.78); 6.85 T1 v T2 <0.001; T1 v T3 <0.001. Also improved confidence in understanding 'the types of intrapartum hypoxia and the resultant features observed on a CTG trace' (p=0.04); 'control of fetal heart (p=0.004)'; 'fetal pathophysiology p=0.005'; however confidence in understanding of 'wider clinical picture' and 'national guidelines' not significant.	
Pettker et al. 2009	Quantitative non-randomised	Educational programme that included dissemination and review of the National Health and Human Development (NICHD) guidelines. This training programme was part of a larger strategy to improve patient safety[4] (Number of participants not stated)	NA (Pre-post)	Obstetricians, midwives, nurses	"Safety Attitudes Questionnaire (SAQ)", a tool adapted from the aviation field and used for the assessment of health care (86).<br><br>Results:<br>2004-2007<br>"good teamwork" 38.5 to 55.4%<br>"good safety climate" 33.3% to 55.4%<br>"good teamwork climate" (nurses) 16.4% to 88.7%	High risk of bias

[4] Multiple safety interventions were incrementally introduced from September 2004 to November 2006 and included outside expert review, protocol standardisation, the creation of a patient safety nurse position and patient safety committee, as well as training in team skills and FHR monitoring interpretation.

					"good teamwork climate" (physicians) 39.5% to 72.7%<br><br>Questionnaire was distributed to all staff involved in obstetric care not just study participants	
Vadnais 2011	Quantitative non-randomised	*A "multifaceted educational approach involving pocket cards, posters, didactic sessions, and an online educational programme on electronic FHR monitoring available to all staff"*<br>(Number of participants not stated, 41 "random" charts reviewed pre and post intervention)	N/A (pre-post)	medical students, nurses, residents, physicians	Outcome:<br>Compliance<br><br>Results:<br>Physician compliance:<br>All components of the FHR tracing and a category: 1 (0.5%) vs. after programme 154 (90%), p<0.001<br><br>Nursing compliance:<br>0 (0%) vs. after programme 149 (87%), p<0.001	High risk of bias
Wagner 2012	Quantitative non-randomised	Multifaceted:<br>Mandatory EFM educational course and exam<br>Team training (STEPPS methodology)<br>Daily teaching<br>Medical records upgrades<br>Standardisation of protocols<br>(Number of participants not stated, but n=217 participated in staff survey in 2007 and N=1731 in 2009)	N/A (pre-post)	Obstetricians, nurses, residents, anaesthesiologists, neonatologists	Outcome:<br>Documentation of fetal heart rate abnormalities<br>"percentage of appropriately managed cases"<br>Staff perceptions of patient safety<br>Patient perceptions "that staff worked together" and "recommend the institution"<br><br>FHR abnormalities documentation:<br>29% (2008) to 100% (2009) [p<0.002]	Low risk of bias

					Appropriate management of FHR abnormalities:<br>53% (2008) to 93% (2009) [p<0.0001]<br>Perception of safety:<br>(Page 10, Figure 2):” positive response” 55% (2007) to 77% (2009)	
Quantitative descriptive						
Burke 2013	Quantitative descriptive	2 hour didactic and simulation session (covered multiple obstetric issues including fetal monitoring)<br>N=372	N/A	Obstetricians, midwives, residents, anaesthesiologists, and nursing staff	“Perceptions of safety” following mandatory training<br><br>“Safety questionnaire” from AHRQ was distributed to all physicians, residents, mid-level providers, and RNs in the hospital in April 2010 (i.e. not only original participants).<br>Repeated in November 2011<br><br>Results:<br>Page 121 Figure 6<br>Fifty-five percent of the labour and delivery respondents selected "excellent" or "very good" as answer to the statement: "Give your unit an overall grade on patient safety." In comparison, the same questionnaire was distributed in November 2011, near the end of the educational session, and 78% scored their	Low risk of bias

					response to the same statement as "excellent" or "very good"." This difference was statistically significant (p=0.003) [2010 N=84; 2011 N=94)	

2.6 Table C8. Maternal/fetal outcomes following CTG training (Kirkpatrick level 4)

Reference	Study design	Training	Comparator	Study participants	Outcome measure	Results	Overall quality assessment
Quantitative non-randomised (or studies reporting pre-post data)							
CTG training only							
Brown et al. 2017	Quantitative non-randomised	EFM one day course complemented by online and hardcopy resource materials	NA (Pre-post)	Maternity care providers	Neonatal mortality (primary outcome), hypoxic ischaemic encephalopathy (HIE) neonatal deaths, NICU admission, respiratory support, APGAR <5 at 5 min, intubation for ventilator support, emergency caesarean rates	Baseline (1998 to 2004) and after intervention (2005 to 2010)<br><br>Term hypoxic intrapartum perinatal deaths reduced from 2.02 to 1.07 per 10,000 total births after 2005 (RR 0.49, 95% CI: 0.35 to 0.68)<br><br>Neonatal mortality amongst babies admitted to the neonatal unit: 14.7% versus 11.2% (RR 0.76, 95% CI: 0.65 to 0.89) (n/N not reported)<br><br>There was no overall decrease in total neonatal deaths (1.5 per 10,000 to 1.6 per 10.000, RR 1.04, 95% CI: 0.88 to 1.23)<br><br>NICU admission for respiratory support or therapeutic hypothermia increased from 10.6 to 14.6 per 10,000 live births (RR 1.37, 95% CI: 1.29 to 1.46)<br><br>The proportion of babies with APGAR <5 at 5 min: 948/1703 (55.7%) versus 1264/2775 (45.5%) (RR 0.82, 95% CI: 0.7 to 0.87)<br><br>Intubation for ventilator support: 938/1703 (55.1%) versus 1275/2775 (45.9%) (RR 0.83, 95% CI: 0.79 to 0.88)	Low risk of bias

						HIE: 615/1703 (36.1%) versus 827/2775 (29.8%) (RR 0.83, 95% CI: 0.76 to 0.90)<br><br>Rates of emergency caesarean section: 117463/1003957* (11.7%) versus 218967/1972676* (11.1%) (RR 0.95, 95% CI: 0.95 to 0.96)<br>*calculated from n and %	
Byford et al. 2014	Quantitative non-randomised	Fetal Surveillance Education Program (FSEP), a book titled 'Fetal Surveillance: A Practical Guide", and an online education programme	NA (Pre-post)	Maternity care staff	Incidence of hypoxic ischaemic encephalopathy (HIE)	Baseline (2003-2005) and after intervention (2006-2011)<br><br>HIE rate was significantly lower after the intervention (p=0.02) (actual rates per year presented in graph form)	High risk of bias
Chandraharan et al. 2014 (Abstract)	Quantitative non-randomised	Use of fetal ECG (ST-Analyser with intensive training and a mandatory competency test). Intensive training was commenced in 2007 and a mandatory test for all staff in fetal monitoring was introduced in 2010.	NA (Pre-post)	Obstetricians and midwives	Emergency caesarean sections; failed instrumental vaginal birth, hypoxic ischaemic encephalopathy (HIE), early neonatal death	Baseline (2008) and after intervention (2012)<br><br>Emergency caesarean section rates: 775*/5167 (15%) versus 342*/3804 (9%) (p<0.0001)<br><br>Failed instrumental vaginal delivery: 40*/5167 (0.77%) versus 27*/3804 (0.71%) (p=0.012)<br><br>HIE was 1.2/1000 in 2008 and 1.1/1000 in 2012 (p ns [value not reported])<br><br>Neonatal death reduced from 1.7/1000 to 1.3/1000 (p ns [value not reported])<br><br>*calculated based on % and N	Unclear risk of bias
Wijemanne 2016 (Abstract)	Quantitative non-randomised	*"an intense physiology-based CTG training and mandatory competency based*	NA (Pre-post)	"all staff" - unclear	Incidence of hypoxic ischaemic encephalopathy (HIE)	26 cases over the entire study period. No pre/post breakdown provided.	High risk of bias

		CTG testing was introduced to all staff.' No further details provided, and number of participants not reported					
Draycott et al. 2006	Quantitative non-randomised	One-day training course on CTG interpretation, with obstetric emergency drill stations	NA (Pre-post)	Midwifery staff, including managerial, community-based and part-time midwives, and all obstetric medical staff, including senior house officers (SHOs), specialist registrars (SpRs) and consultants	Emergency caesarean sections, 5-minute Apgar ≤6, hypoxic ischaemic encephalopathy (HIE), Moderate/severe HIE	Baseline (1988-1999) and after intervention (2001-2003)<br><br>Emergency caesarean section rates: 784/8430 (9.3%) versus 1254/11030 (11.4%) (p<0.001)<br><br>5-minute Apgar ≤6: 73/8430 (0.87%) versus 49/11 030 (0.44%), RR 0.51 (95% CI: 0.35, 0.74)<br><br>HIE: 23/8430 (0.27%) versus 15/11030 (0.14%), RR 0.50 (95% CI: 0.26, 0.95)<br><br>Moderate/severe HIE: 16/8430 (0.19%) versus 11/11030 (0.10%), RR 0.53 (95% CI: 0.24, 1.13)	Low risk of bias
Thellesen et al. 2017 / Thellesen et al 2019	Quantitative nonrandomised (the authors described the 2017 study as a cross-	The implementation of a national CTG education programme (1-day course) (n=1641, but pre-test/post-test data based on n=790)<br><br>Patient/delivery outcomes (Thellesen 2019)	NA (pre-post)	Gynaecologists, obstetricians, general specialists, residents, midwives	10-item CTG multiple choice test (to test knowledge, interpretation skills and decision-making)	Outcome measure: % of participants who improved their test scores<br><br>Baseline test (Test 1): 790/1641 (48.1%)* had test scores from 0-9<br><br>Immediately after intervention (Test 2):<br>The authors appear to report that of 790 course participants who scored 0-9 on the test before implementation, 662 (83.8%) improved their score in the post-test (immediately after	Low risk of bias

	sectional study, but pre-post data were also reported)	All intended vaginal deliveries in Denmark resulting in a liveborn singleton in ce-phalic presentation with a gestational age≥37weeks.				the course) (no statistical comparisons were reported)<br><br>*data calculated from figures reported in the paper<br><br>Patient/delivery outcomes (Thellesen 2019)<br><br>Pre-implementation; post-impl; 3 month follow-up:<br><br>5 min Apgar score<7: ref; 0.97(0.84, 1.11); 0.99 (0.90, 1.10);<br>Emergency caesarean: ref; 1.05 (1.01, 1.08); 0.98 (0.96, 1.01)<br>Assisted vaginal delivery: ref; 0.91 (0.87-0.95); 0.86 (0.84,0.89);<br>Umbilical cord pH <7.00 and neonatal therapeutic hypothermia ns at both time periods.	
Ting 2017	Quantitative non-randomised	1-hour session (30min lecture, case base discussion and video demonstration). Employing the Situation-background-assessment-recommendation (SBAR) technique(91) (n=3358 pre-intervention and 1346 post)	NA (Pre-post)	nurses	Apgar score <7 at 5 minutes	Preintervention 145/3368 (4.3)<br>Postintervention 60/1346 (4.5)	High risk of bias
Katsuragi et al. 2015	Quantitative non-	Weekly training for 6 months to educate staff in the rule-based	NA (Pre-post)	Physicians and nurses	Umbilical arterial acid base and blood gas data in case of	Baseline (2003-2004) and after intervention (2006-2007)	High risk of bias

	randomised	management of FHR patterns and National Institute of Child Health and Human Development FHR pattern descriptions			variable decelerations 10 min before delivery	Umbilical arterial acid base and blood gas:<br>pH: 7.29 (SD 0.11) versus 7.36 (SD 0.13), p<0.05<br>Base Excess (mmol/L): -6.6 (SD 1.2) versus -2.8 (SD 0.9), p<0.05<br>Umbilical arterial pO2 (mmHg): 19.5 (SD 2.1) versus 21.92 (SD 3.1), p<0.05<br>Umbilical arterial pCO2: 43.33 (SD 12.5) versus 38.47 (SD 9.6), p<0.05<br><br>The authors also reported that the "adoption of the management system was associated with significant decreases in variable decelerations before versus after the introduction of the management system at 60, 50, 40, 30, 20 and 10 minutes before vaginal delivery."	
Quantitative descriptive							
Al-Sammarai 2019 (Abstract)	Quantitative descriptive	QI initiative at Sherwood Forest Hospitals to enhance CTG training and situational awareness; to enable all midwifery and core medical staff to access physiologically based CTG training. Clinical leaders were also given the opportunity to attend advanced training sessions. This training supported enhanced assessment	None	All midwifery, core medical staff and clinical leaders	"HIE reportable cases to Each Baby counts";	No Reportable cases since July 2017; HIE 1-3 rate in 2018 "dropped by 85% compared to 2016.	Unclear risk of bias

		and decision-making. Additionally, since 2016, training has focused on human factors, allowing better understanding of why incidents occur and ways their effects can be ameliorated.					
CTG training as part of wider organisational changes							
Blomberg 2016	Quantitative non-randomised (*Pre-post comparisons reported)*	Complex organisational change over several years[5] (one element was CTG course plus an exam which was introduced in 2009)	NA (Pre-post)	Not reported - organisational level (delivery unit)	Caesarean section rates; rate of instrumental vaginal deliveries; prevalence of new-borns with umbilical cord pH <7 and APGAR score <4 at 5 min	Baseline (2006) and after intervention (2014)<br><br>Caesarean section rates (both emergency and elective): 529/2700 (20%) versus 312/2936 (10%) (p< 0.001)<br><br>Overall rate of instrumental vaginal deliveries: 10.1% (n/N not reported) versus 5.5% (p not reported)<br><br>Prevalence of new-borns with umbilical cord pH <7 and APGAR score <4 at 5 min: The authors reported that they "were the same over the years studied."	High risk of bias

[5] Nine items were introduced and established at different times during a 10-year period: Item 1 (monitoring of obstetric results) was introduced in late 2006 and items 2 (recruitment of a midwife coordinator), 3 (risk classification of women), 6 (obstetrical morning round), and 9 (public promotion of the strategy) in 2007. Item 4 (three midwife competence levels) was introduced stepwise and fully developed to its present form in 2012. Item 7 (fetal monitoring skills) was established in 2009 and item 8 (obstetrical skills training) was introduced in a premature form in 2008 and fully implemented in its present form with scenarios and debriefing in 2010.

						The authors also reported that "between 2009 and 2013, the overall rate of HIE grade I–III in our unit was 1.47/1000 children born (20/13 622). The comparable rate in Sweden during the same period was 1.32/1000 (743/561 840) (p=0.64)."	
Sibanda 2009	Quantitative non-randomised	*"a CTG education program, as well as the immediate implementation of a mentorship scheme for all newly qualified members of staff working on the labour ward"* As part of a wider risk management system and Root Cause Analysis (RCA) review of safety incidents (Number of participants not stated)	NA (Pre-post)	Unclear	Low Apgar score (<7)	Preintervention 23/3965 (0.58%)<br>Postintervention 17/3763 (0.45%)	Low risk of bias
Goffman et al. 2014	Quantitative non-randomised	On-line electronic fetal monitoring course plus a test, team training and one-day simulation education course – (which were variously introduced after 2008[6])	NA (Pre-post)	Obstetricians, midwives, nurses, residents	Overall adverse outcome index (AOI) (percentage of deliveries affected by one or more adverse events); Weighted Adverse Outcome Score	Baseline (January to June 2008) and after intervention (July to December 2011)<br><br>Overall AOI: 26*/2445 (10.7%) versus 147*/2368 (6.2%) (p<0.001)<br><br>*calculated based on % and N	High risk of bias

[6] Before 2008, changes to the hospitals involved included:

		In addition to these educational interventions, one other change occurred: The use of enhanced electronic medical records (in 2010)			(WAOS) (the sum of the points assigned to cases with adverse outcomes), neonatal death, APGAR <7 at 5 minutes	WAOS: 3.9 versus 2.3 (p=0.001)<br><br>There were no intrapartum or neonatal deaths in 2008 and in 2011<br><br>APGAR <7 at 5 minutes: 15/2445 (0.61%) versus 12/2368 (0.51%) (p=0.62)	
Pettker 2009	Quantitative non-randomised	Educational programme that included dissemination and review of the National Health and Human Development (NICHD) guidelines. This training programme was part of a larger strategy to improve patient safety[7]	NA	Obstetricians, midwives, nurses	Adverse events as measured using the Adverse Outcome Index (AOI) (the number of deliveries with associated adverse events per total deliveries for each 3-month period)	Baseline (September 2004) with quarterly review periods up to August 2007<br><br>The authors reported "The mean quarterly AOI for the first half of the initiative (2.90 +- 0.64%) was also significantly different than that for the second half (2.09 +- 0.57%) (Student t test, P=.04)."<br><br>*The EFM certification was given at the 6th quarter (which was in the middle of the study initiative)*	High risk of bias
Wagner et al. 2011	Quantitative non-random	EFM education on-line course and exam (as part of a wider set of interventions[8])	NA (Pre-post)	'The entire perinatal team'	Adverse events as measured using the Modified Adverse Outcome Index (AOI)	Baseline (2007) and eight review periods (four in 2008 and four in 2009)	Low risk of bias

a. Dissemination of best practice guidelines (November 2007)
b. Standardized education - involving a grand rounds curriculum that was presented to members of Obstetrics and Gynaecology and allied departments, which were used to make it clear that compliance with guidelines was expected, and to highlight upcoming interventions. In addition, monitoring with ongoing audit and feedback approach was started in 2008.

[7] Multiple safety interventions were incrementally introduced from September 2004 to November 2006 and included outside expert review, protocol standardisation, the creation of a patient safety nurse position and patient safety committee, as well as training in team skills and FHR monitoring interpretation.

[8] Other safety initiatives incrementally introduced from 2007 were the use of STEPPS methodology for team training, and daily multidisciplinary teaching rounds, enhancement of medical records, the introduction and standardisation of protocols, and an obstetrical emergency simulation programme in high-risk care scenarios.

	ised *(Pre-post comparisons reported)*				(assessed by reviewing all obstetrical haemorrhage charts and 10 charts with abnormal FHR tracings each month)	The Modified AOI rate: 1.92% at baseline versus 0.89% in last review period. The odds ratio was 0.46 (confidence intervals were not reported) for a comparison between these two years<br><br>The authors reported "specifically, the odds ratio for the first quarter in 2008 was 0.768 (i.e. a 23% decrease in MAOI risk) and decreased to 0.291 (i.e. a 71% decrease in risk) for the first quarter in 2009, levelling at about 0.460 (i.e. a 54% decrease) for the last three quarters of 2009.	
Simpson 2009	Quantitative non-randomised	Fetal monitoring training (called 'fetal assessment' in the article) was one of various components of the patient safety program. It included face-to-face educational sessions (review process) using medical records as the basis. For the FHR evaluation component: review courses and study materials included textbooks (Number of participants not reported)	NA (Pre-post)	Unclear	Birth trauma rates<br>Obstetrical occurrences<br>Obstetrical claims<br>Numbers participating in fetal monitoring<br>Algorithm use<br>Audit participation	Regarding the fetal monitoring component of the program, only process measures are reported: between the initial assessment and the first follow-up evaluation, the number of hospitals implementing interdisciplinary fetal monitoring education went from 3 to 16 (19% to 100%); use of clinical algorithm for intrauterine resuscitation went from 9 to 16 hospital (56% to 100%); medical record documentation forms and electronic systems with cues for use of NICHD definitions increased from 3 hospital to 13 (from 19% to 81%); and hospitals doing medical record audits including the fetal monitoring strips went from 2 to 13 (13% to 81%).	High risk of bias
Young et al. 2001	Quantitative non-random	Six training sessions in cardiotocography, given over two half days every six months	NA (Pre-post)	Midwives and physicians	The number of babies who received suboptimal care (critical	Baseline (Oct 1994) and five audit periods (Oct/Nov 1995, Dec 1995/Sept 1996, Aug/Dec 1997, Jan/July 1998, and Aug 1998/Jan 1999)	High risk of bias

	ised *(Pre-post comparisons reported)*	(as part of a wider set of interventions[9])			retrospective analysis of case notes by a team to determine whether or not there was evidence of suboptimal care)	Grade II/III suboptimal care: 14/19 (74%) versus "after CTG training was made compulsory" (i.e. the last audit period): 9/96 (9.3%)<br><br>The authors reported that "differences seen in overall distribution of [Confidential Enquires into Stillbirths and Deaths in Infancy] CESDI scores were highly significant according to the Kruskal-Wallis statistic (based on chi squared=46.23)."	
Richardson 2018 (Abstract only)	Quantitative non-randomised	Multidisciplinary emergency obstetric training - simulated scenarios relating to pathological CTG scenarios as one component of much wider training.	NA (pre-post)	Not reported	Major obstetric haemorrhage<br>APGAR scores < 7<br>Incidence of category 1 sections and shoulder dystocia	Incidence of major obstetric haemorrhage increased by 22%.<br>Incidence of APGAR scores <7 fell by 16%.<br>incidence of category 1 sections and shoulder dystocia reduced by 1.3% and 17%	Unclear risk of bias
Ebenezer 2019	Quantitative non-randomised	Series of quality improvement interventions conducted over 18 years including training in CTG (in 2003). However, very wide range of interventions also introduced, including CTG training for all	NA (pre-post, retrospective cohort study)	Whole maternity unit	Main outcome measures were perinatal mortality rate, birth asphyxia rate, and caesarean section rate.	The perinatal mortality rate has decreased from 44/1000 births in the year 2000, to 16.4/1000 births by the year 2018, which was highly significant (P < 0.001). Babies born with birth asphyxia requiring admission to the neonatal unit decreased from 24/1000 in 2001 and 6/1000 in 2009 to 0.7/1000 in 2018, P < 0.00001.	Unclear risk of bias

[9] Other interventions included: local guidelines, regular monthly audit, and monthly feedback meetings.

		women in labour, continuous monitoring for all women receiving oxytocin and rigorous perinatal audits. Cannot separate the effect of CTG training alone.					
