# Supplementary material for: Training in the use of intrapartum electronic fetal monitoring with cardiotocography: systematic review and meta‐analysis
Source: BJOG. 2021 Jan 22;128(9):1408–19. doi: 10.1111/1471-0528.16619 (PMC8359372; doi:10.1111/1471-0528.16619)
Supplement: Supplementary file 6 — Appendix S4. GRADE assessment / summary of findings table Setting: maternity care Contents [file BJO-128-1408-s013.pdf]

Appendix S4. GRADE assessment / summary of findings table Setting: maternity care

Contents

1.1 Question: CTG training compared to other types of training, or different components of training for CTG interpretation and management 2

1.2 Question: CTG training compared to no training, or before and after training for CTG interpretation and management 5

1.3 Question: CTG training as part of much wider organisational changes compared to no training 2

1.1 Question: CTG training compared to other types of training, or different components of training for CTG interpretation and management

CI: Confidence interval; SMD: Standardized mean difference

Key:

Certainty assessment							№ of patients		Effect		Certainty	Importance
№ of studies	Study design	Risk of bias	Inconsistency	Indirectness	Imprecision	Other considerations	CTG education	usual training	Relative (95% CI)	Absolute (95% CI)		
Test Scores (Training A v Training A+) - RCT evidence												
4	randomised trials	serious	serious b	very serious a	serious c	none					⊕◯◯◯ VERY LOW	
Test Scores (Training A v Training B) - RCT Evidence												
2	randomised trials	very serious d	not serious	not serious	serious c	none					⊕◯◯◯ VERY LOW	
Test Scores (Training A v Training B) - Non randomised evidence												
3	observational studies	serious e	serious b	serious a	serious c	none					⊕◯◯◯ VERY LOW	

a. heterogeneity of intervention types
b. Inconsistency in the direction of effect across trials
c. Insufficient optimal information size
d. All studies rated as high risk of bias
e. >50% of studies reported as high or unclear risk of bias

1.2 Question: CTG training compared to no training, or before and after training for CTG interpretation and management

Certainty assessment							№ of patients		Effect		Certainty	Importance
№ of studies	Study design	Risk of bias	Inconsistency	Indirectness	Imprecision	Other considerations	CTG education	usual training	Relative (95% CI)	Absolute (95% CI)		
Test Scores (Training v No Training) - RCT evidence												
5	randomised trials	serious a	not serious b	not serious c	serious	none			-	SMD 0.91 SD higher (0.47 higher to 1.34 higher)	⊕⊕◯◯ LOW	
Test Scores (Training v No Training) - Non randomised evidence												
13	observational studies	serious d	not serious	serious c	serious e	none					⊕◯◯◯ VERY LOW	
Inter-observer agreement (Training v no training)												
4	observational studies	serious d	not serious	serious c	serious g	none					⊕◯◯◯ VERY LOW	
Participant performance (Training v No Training)												
3	randomised trials	not serious	not serious f	serious e	serious g	none					⊕⊕◯◯ LOW	
Behaviour change and application of learning												
8	observational studies	serious d	not serious	not serious	very serious g	none					⊕◯◯◯ VERY LOW	
Hypoxic ischaemic encephalopathy (HIE) - CTG Training alone												
6	observational studies	serious d	not serious	not serious	not serious	none					⊕◯◯◯ VERY LOW	
Emergency caesarean sections - CTG Training alone												
4	observational studies	serious j	very serious f	not serious	not serious	none					⊕◯◯◯ VERY LOW	
APGAR (<5; <=6; 7) at 5 minutes - CTG Training alone												
4	observational studies	serious j	not serious	not serious	not serious	none					⊕◯◯◯ VERY LOW	
Neonatal deaths - CTG Training alone												

2	observational studies	serious j	not serious	not serious	not serious	none		⊕◯◯◯ VERY LOW	

CI: Confidence interval; SMD: Standardized mean difference

Key:

a. All studies were rated as high or unclear risk of bias

b. statistical heterogeneity (e.g. high i squared)

c. heterogeneity of intervention types

d. All most all studies reported high or unclear risk of bias

e. heterogeneity of intervention and outcome types

f. Inconsistency in the direction of effect across trials

g. Insufficient optimal information size

h. All studies rated as high risk of bias

i. 50% of studies reported as high risk of bias

1.3 Question: CTG training as part of much wider organisational changes compared to no training

CI: Confidence interval; SMD: Standardized mean difference

Certainty assessment							№ of patients		Effect		Certainty	Importance
№ of studies	Study design	Risk of bias	Inconsistency	Indirectness	Imprecision	Other considerations	CTG education	usual training	Relative (95% CI)	Absolute (95% CI)		
Adverse Outcome Index (AOI) - CTG training as part of wider organisational changes												
3	observational studies	very serious a	not serious	not serious	not serious	none					⊕◯◯◯ VERY LOW	

Key:

a. All studies were rated as high or unclear risk of bias
