# Supplementary material for: Training in the use of intrapartum electronic fetal monitoring with cardiotocography: systematic review and meta‐analysis
Source: BJOG. 2021 Jan 22;128(9):1408–19. doi: 10.1111/1471-0528.16619 (PMC8359372; doi:10.1111/1471-0528.16619)
Supplement: Supplementary file 7 — Appendix S5. Summary risk of bias for individual studies. [file BJO-128-1408-s004.pdf]

Appendix S5. Summary risk of bias for individual studies

1.1 Figure S5.1. Summary risk of bias for individual studies: quantitative, randomised controlled trials (MMAT criteria)

	**1**	**2**	**3**	**4**	**5**	**6**
Beckley 2000	Unclear	Unclear	Low	Unclear	Unclear	Unclear
Carbonne 2016	Low	Low	High	High	Unclear	High
Devane 2006	Low	High	Low	Unclear	Low	High
Evans 1998	Low	Low	Low	High	Low	Low
Fransen 2013	Low	Low	Low	Low	Low	Low
Kinnick 1990	High	Unclear	Low	Unclear	Unclear	High
Murray 1996	Unclear	Unclear	High	Unclear	Unclear	High
Rizk 2013	Unclear	Low	Low	Unclear	Unclear	Unclear
Trepanier 1996	Unclear	Low	Low	Unclear	Unclear	Unclear
Wilson 1998	Unclear	High	Low	Unclear	Low	High
Wilson 2000	Unclear	Unclear	High	Unclear	Unclear	High
Wilson 2001	Unclear	Unclear	High	Unclear	Unclear	High
Cuerva 2017	High	Unclear	Low	High	Low	High

<u>MMAT criteria: Quantitative, Randomised</u>

1. Is randomization appropriately performed?
2. Are the groups comparable at baseline?
3. Are there complete outcome data?
4. Are outcome assessors blinded to the intervention provided?
5. Did the participants adhere to the assigned intervention?
6. Summary risk of bias estimate

1.2 Figure S5.2. Summary risk of bias for individual studies: quantitative, non-randomised studies (MMAT criteria)

	1	2	3	4	5	6
Ayers-de-Campos 2004	High	High	Low	Low	Unclear	High
Chandraharan 2014	Unclear	Low	Unclear	Unclear	Unclear	Unclear
Blomberg 2016	High	High	Unclear	Low	Unclear	High
Brown 2017	Low	Low	Unclear	Low	Unclear	Low
Byford 2014	Low	High	Low	Low	Unclear	High
Cook 2015	Unclear	Unclear	Low	Unclear	Unclear	Unclear
Cooke 2010	Unclear	Unclear	Unclear	Unclear	Unclear	Unclear
Daglar 2019	Low	Low	Low	High	Low	Low
Davis 2010	Unclear	High	Unclear	Unclear	High	High
Di Lieto 2002	Unclear	Unclear	Unclear	Unclear	High	High
Draycott 2006	Low	Low	Low	Low	Low	Low
Ebenezer 2019	Low	Low	Low	High	Unclear	Unclear
Froc 2018	Low	Low	High	Unclear	Unclear	Low
Gnanasambanthan 2018	Unclear	High	Unclear	High	Unclear	High
Goffman 2014	Unclear	Low	Low	Unclear	High	High
Govindappagari 2016	Unclear	Unclear	Unclear	Low	Low	Unclear
Grace 2018	High	Unclear	Unclear	Unclear	Unclear	High
Gyllencreutz 2017	Low	Low	Low	Low	Low	Low
Jomeen 2019	Low	Low	Unclear	Unclear	Low	Unclear
Katsuragi 2015	Unclear	Low	Low	High	Low	High
Lee 2019	Unclear	Low	Low	Low	High	Unclear
Keegan 2016	Low	Unclear	High	Low	Unclear	High
MacEachin 2009	Unclear	Low	High	High	Low	High
Mahley 1998	Unclear	Unclear	N/A	High	Unclear	High
Millde-Luthander 2012	Low	Low	High	Low	Unclear	High
Miller 2013	Low	Low	Low	High	Unclear	High
O’Boyle 1995	Low	Low	Low	Low	Unclear	Low
Parsons 2013	High	Unclear	Low	High	Unclear	High
Pettker 2009	Unclear	Low	Low	High	High	High
Pettker 2011	Unclear	Low	High	Unclear	Low	High
Richardson 2018	Unclear	Unclear	Unclear	Unclear	Unclear	Unclear
Sibanda 2009	Unclear	Low	Low	High	Unclear	Low
Simpson 2009	Unclear	High	Low	High	High	High
Stohl 2016	High	Low	Unclear	High	Unclear	High
Thellesen 2017/2019	Low	Low	Low	Low	Low	Low
Ting 2017	Low	Low	Low	High	Low	High
Vadnais 2011	High	High	Low	High	Low	High
Wagner 2012	Unclear	Low	Low	Low	Unclear	Low
Wijemanne 2016	Unclear	Unclear	High	High	High	High
Young 2001	Unclear	High	Unclear	High	Unclear	High

<u>MMAT criteria: Quantitative, Non-Randomised:-</u>

1. Are the participants representative of the target population?
2. Are measurements appropriate regarding both the outcome and intervention (or exposure)?
3. Are there complete outcome data?
4. Are the confounders accounted for in the design and analysis?
5. During the study period, is the intervention administered (or exposure occurred) as intended?
6. Summary risk of bias estimate

1.3 Figure S5.3. Summary risk of bias for individual studies: quantitative descriptive studies (MMAT criteria)

	1	2	3	4	5	6
Blix 2005	Low	Unclear	High	High	High	Low
Burke 2013	Unclear	Unclear	Low	Unclear	Unclear	Low
Catanzarite 1987	Unclear	High	Low	Unclear	Low	Low
Guild 1994	Unclear	High	High	High	Low	Low
Haire 1978	Unclear	Unclear	High	Unclear	High	Unclear
Kroushev 2009	High	High	High	High	Unclear	High
Rehling-Anthony 2011	High	High	High	Unclear	N/A	High
Ren-He-Ya 2017	High	Unclear	High	High	High	High
Volaw 1979	Low	Low	High	Low	Low	Unclear
Al-Samarrai 2019	Unclear	Unclear	Unclear	Unclear	Unclear	Unclear
Knight 2019	Unclear	Unclear	Unclear	Unclear	High	High

<u>MMAT criteria: Quantitative descriptive:-</u>

1. Is the sampling strategy relevant to address the research question?
2. Is the sample representative of the target population?
3. Are the measurements appropriate?
4. Is the risk of nonresponse bias low?
5. Is the statistical analysis appropriate to answer the research question?
6. Summary risk of bias estimate
