# Supplementary material for: Training in the use of intrapartum electronic fetal monitoring with cardiotocography: systematic review and meta‐analysis
Source: BJOG. 2021 Jan 22;128(9):1408–19. doi: 10.1111/1471-0528.16619 (PMC8359372; doi:10.1111/1471-0528.16619)
Supplement: Supplementary file 8 — Appendix S6. Cardiotocography training approaches used in included studies. [file BJO-128-1408-s017.pdf]

Appendix S6. CTG training approaches used in included studies

CTG training approaches used in included studies	
Training approach	Studies using approach and further details
Computer aided	Ten studies used “computerised” or “computer-aided” learning to instruct teaching (Di Lieto 2002, Guild 1994, Murray 1996, Beckley 2000, Votaw 1979, Wilson 1998, Wilson 2000, Wilson 2001, Catanzarite 1987).<br>One recent study employed a mobile application to supplement class work (Keegan 2016).
e-learning	Six, more recent, studies specified the use of online eLearning resources and assessment (either alone or in addition to face to face teaching) to both teach and ‘certify’ learners (Carbonne 2016, Govindappagari 2016, Gyllencreutz 2017, MacEachin 2009, Thellesen 2017/2019, Pettker 2009).
Classroom, tutorials and workshops	Traditional classroom work was described by five studies (Trepanier 1996, Blix 2005, O’Boyle 1995, Mahley 1999, Draycott 2006, Jomeen 2019, Daglar 2019, Froc 2018), with some adding additional features e.g. ‘videotape’ instruction (O’Boyle 1995), “process cards” to guide in rhythm analysis (Mahley 1999). Two studies explicitly stated teaching provided was one to one (Draycott 2006, Jomeen 2019). Two studies used a combination of classroom and practical sessions (Daglar 2019, Froc 2018). One study described a structured education programme with weekly CTG masterclasses (Gnanasambanthan 2018), but little further information was available (abstract only available). One study reported an interactive workshop (Grace 2018) but little further information was available (abstract only available). One study reported multidisciplinary teaching but little further information was available (abstract only available).
Lecture	Traditional lectures and/or implementation of novel teaching concepts to convey information were a feature of five studies (Cook 2015, Devane 2006, Kinnick 1990, Ting 2017, Sibanda 2009).
Simulation	Simulation models and skills based training were a feature of eight studies (Rizk 2013, Lee 2019, Fransen 2013, Cuerva 2018, Ren 2017, Burke 2013, Davis 2010, Richardson 2018).
Chart audit and feedback	Auditing of chart and targeting ‘education as required’ was employed by one study (Davis 2010).
Diagnostic criteria/Algorithms.	Three studies evaluated the impact of teaching and adhering to strict diagnostic criteria or algorithms in CTG interpretation (Katsuragi 2015, Ayres-de-Campos 2004, Stohl 2016).
Train the trainer	Two studies referred to ‘train the trainer’ instruction but again failed to provide comprehensive detail on the content/method of instruction (Cooke 2010, Miller 2013).
Multifaceted interventions	Three studies specifically evaluated the impact of a ‘National Fetal Surveillance Education Programme’ with elements of classroom work, online resources and ‘refresher courses’ (Byford 2014, Kroushev 2009, Brown 2017).<br>Two studies provided ‘mentorship’ and a ‘CTG education programme’ prompted by risk management system alerts (Sibanda 2009, Simpson 2009).<br>Other studies had multiple elements targeting EFM training including courses, team training and simulation (Goffman 2014); guideline dissemination, review sessions, ‘allocation of study guides’ and certification (Pettker 2009, Pettker 2011, Wagner 2012, Young 2001), eLearning and ‘pocket cards’ (Vadnais 2011).<br>One study reported CTG training with additional support in advanced assessment and decision making plus human factors training (Al-Samarrai 2019) but little further information was available (abstract only available)<br>Studies that included fetal monitoring skills as one element of larger complex organisational changes to improve patient safety are shown in the Full data tables (Appendix S3)

References

Full study references are in the reference list of the full paper.
