# Supplementary material for: Training in the use of intrapartum electronic fetal monitoring with cardiotocography: systematic review and meta‐analysis
Source: BJOG. 2021 Jan 22;128(9):1408–19. doi: 10.1111/1471-0528.16619 (PMC8359372; doi:10.1111/1471-0528.16619)
Supplement: Supplementary file 9 — Appendix S7. Crowd contributors. [file BJO-128-1408-s009.pdf]

Appendix S7. Crowd contributors

We thank the Cochrane Crowd contributors listed below for their support of the review through their involvement in additional methodological development work around screening decisions embedded in this review:-

Nikolaos Sideris, Arulmani Thiyagarajan, Richard James, Luma Haj Kasem, Karen Ma, Susi Wisniewski, Jacques Jansen van Vuuren, Therese Dalsbø, Hariklia Nguyen, Sue Faulds, David Sullivan, Jo Thompson Coon, Joyce Coutu, Ngoc-Minh Nguyen, Stella Maria O'Brien, Danial Sayyad, Max Strube, Simon Stones, Riccardo Guarise, Robert Robinson, Sarah Bruch, Brady Catherine, Vanisree Staniforth, Praveen Hoogar, James Matthews, Tomislav Meštrović, Chun Yu Yeung, Donald Bourne, Katarina Paunovic, Vittoria Lutje, Louise Murphy, Ali Tafazoli, Nai Ming Lai, Ahmed Montasr, Nyuk Jet Chong, Bovey Wu, Peter Davidson, Anna Maria Paloma Lohikko, Sze Wah Samuel Chan, Andrew Ying, Nicole Edworthy, Li Khim Kwah, Gochi Nwulu, John Beddoe, Nicole Askin, Patricia Dwyer-Hallquist, Sebastian Ortiz, Mary MacCara, Aleksandra Pelczarska, Cecily Gilbert, Mhd Wafa Alimam, Nurun Nisa de Souza, Alexandra McAleenan, Michael Capraro, Celia Almeida, Maura Scott, Esteban González, Nuno Fernandes, Caroline Struthers, Nicolas Vinay, Leonardo Perales Guerrero, Emmet Farragher, Ghaleb Muhammad Mehyar, Sergiu Chirila, Kris Noble, Emanuele Crocetti, Ruth Suhami, Kaloyan Kaloyanov, David Stopforth, Igor Svintsitskyi, Brian Li, Jennie Ver Steeg, Thomas Rosengren, Václav Löffelmann, Adeola Ajayi, Rachel Playforth, Seona Hamilton, Frank Sandmann, Allen McLean, Mgolsorkhi Golsorkhi, Elena Lantsova, Elvira van Dalen, Alexandru Enachioaie, Benjamin Uribe Cota, Karina Pintson, Valerie Wells, Domenico Benvenuto, Merethe Kumle, G Jayapradha, Denise Vitalone, Hebatullah Abdulazeem, Titilope Akinola, Tamer Aboushanab, Aoife O'Mahony, Dorothy Halfhide, Lynn Hampson, Amanda Qiao Ying Yap, Emily Senerth, Tineke Crawford, Sunu Alice Cherian.
